# Identification of Recurrent Variants in *BRCA1* and *BRCA2* across Multiple Cancers in the Chinese Population

**DOI:** 10.1155/2020/6739823

**Published:** 2020-08-15

**Authors:** Yue Jiang, Ting Tian, Chengxiao Yu, Wen Zhou, Junzhe Yang, Yifeng Wang, Yang Wen, Jiaping Chen, Juncheng Dai, Guangfu Jin, Hongxia Ma, Hongbing Shen, Zhibin Hu

**Affiliations:** ^1^State Key Laboratory of Reproductive Medicine, Nanjing Medical University, Nanjing, Jiangsu 211100, China; ^2^Department of Epidemiology, Center for Global Health, School of Public Health, Nanjing Medical University, Nanjing 211166, China; ^3^Jiangsu Key Lab of Cancer Biomarkers, Prevention and Treatment, Collaborative Innovation Center for Cancer Personalized Medicine, Nanjing Medical University, Nanjing 211166, China; ^4^Department of Breast Surgery, The First Affiliated Hospital of Nanjing Medical University, Nanjing, Jiangsu 210029, China

## Abstract

*BRCA1* and *BRCA2* as important DNA repair genes have been thoroughly investigated in abundant studies. The potential relationships of *BRCA1/2* pathogenic variants between multicancers have been verified in Caucasians but few in Chinese. In this study, we performed a two-stage study to screen *BRCA1/2* pathogenic variants or variants of uncertain significance (VUS) with 7580 cancer cases and 4874 cancer-free controls, consisting of a discovery stage with 70 familial breast cancer cases and a subsequent validation stage with 7510 cases (3217 breast cancer, 1133 cervical cancer, 2044 hepatocellular carcinoma, and 1116 colorectal cancer). 48 variants were obtained from 70 familial breast cancer cases after *BRCA1/2* exon detection, and finally, 20 pathogenic variants or VUS were selected for subsequent validation. Four recurrent variants in sporadic cases (*BRCA1* c.4801A>T, *BRCA1* c.3257del, *BRCA1* c.440del, and *BRCA2* c.7409dup) were identified and three of them were labeled Class 5 by ENIGMA. Two variants (*BRCA1* c.3257del and c.440del) were specific in breast cancer cases, while *BRCA2* c.7409dup and c.4307T>C were detected in two hepatocellular carcinoma patients and the *BRCA1* c.4801A>T variant in one cervical cancer patient, respectively. Moreover, *BRCA1* c.3257del was the most frequent variant observed in Chinese sporadic breast cancer and showed increased proliferation of *BRCA1*^c.3257del^-overexpressing triple-negative breast cancer cell lines (MDA-MB-231) in vitro. In addition to the known founder deleterious mutations, our findings highlight that the recurrently pathogenic variants in breast cancer cases could be taken as candidate genetic screening loci for a more efficient genetic screening of the Chinese population.

## 1. Introduction

Breast cancer is considered the most common cancer among females, with approximately 1.7 million new cases worldwide in 2012, accounting for 25% of all new cancer cases in women [1]. As a complex disease, the development of breast cancer is influenced by environmental and genetic factors. It is estimated that 5%-10% of breast cancer cases in women are associated with hereditary susceptibility due to pathogenic variants in some tumor suppressor genes [2]. To date, *BRCA1*, *BRCA2*, *PTEN*, *ATM*, and *CHEK2* have been sequentially reported as medium-to-high penetrant genes associated with breast cancer risk [3]. Among these genes, *BRCA1* and *BRCA2*, as well-known tumor suppressor genes, were associated with breast cancer risk, which was identified from 1994 to 1995 [4, 5]. Previous population studies have identified several *BRCA1/2* pathogenic mutations in Caucasians, for example, *BRCA1* c.68_69del, *BRCA1* c.5266dup, and *BRCA2* c.5946del were the most frequent variants and could greatly increase the cumulative risk of breast cancer during a woman's lifetime [6–10]. Based on these susceptibility loci, genetic testing was widely popularized in Europe and North America, and customized prevention or clinical health management was recommended according to the individual testing results, which could provide an opportunity to reduce the breast cancer risk for those *BRCA1/2* pathogenic variant carriers. However, these *BRCA1/2* variant spectra from Caucasians might be inapplicable in the Chinese population due to the different genetic backgrounds. In addition, people from high-risk groups will have “negative” results, possibly due to unidentified variants, which would present a dilemma for risk assessment and genetic counseling [11].

Since most studies have limited their observations of *BRCA1/2* only in breast and ovarian cancer, the potential relationship of *BRCA1/2* pathogenic variants with other cancer types might be underestimated. Early in 2002, a cohort study containing 11,847 individuals from 699 families of the Breast Cancer Linkage Consortium (BCLC) found that *BRCA1* pathogenic mutations might increase the risk of abdominal cancers in women or pancreatic cancer in men [12]. More recently, a systematic analysis of 10,389 cases of 33 cancer types from The Cancer Genome Atlas (TCGA) showed that *BRCA1/2* genes were the most enriched genes with pathogenic or like pathogenic variants across multicancer types, and some cancers shared the recurrent variant *BRCA1/2* of breast cancer [13]. These findings suggest that as important DNA repair genes, *BRCA1/2* might participate in a certain universal biological process to influence the development of other cancer types in addition to breast and ovarian cancers. Therefore, we hypothesized that the recurrent or case-only *BRCA1/2* variants from familial breast cancer patients might be considered as susceptibility loci for high-risk population screening. We aimed to accurately evaluate the prevalence of case-only variants in *BRCA1/2* not only in breast cancer cases but also for other cancer types (e.g., cervical cancer, colorectal cancer, and hepatocellular carcinoma). Consequently, we conducted a two-stage study to systematically describe the *BRCA1/2* spectra of Chinese women by direct sequencing in familial breast cancer patients and validated the candidate variants in multicancer-type samples.

## 2. Materials and Methods

### 2.1. Ethics Statement

This study was approved by the ethical committees of Nanjing Medical University, China. All participants provided written informed consent.

### 2.2. Patient Samples

We conducted a two-stage study, including a discovery stage (case-only study) and a validation stage (case-control study) to systematically investigate the case-only *BRCA1/2* variants in Chinese population and validate the variants among other cancers. In the discovery stage, a total of 70 breast cancer cases with family histories were included with the following criteria: cases with one or more first- or second-degree relatives affected with breast cancer and/or ovarian cancer. The validation stage contained sporadic cancer cases of breast cancer without family history, cervical cancer, hepatocellular carcinoma, colorectal cancer, and cancer-free controls. All cancer cases were histopathologically confirmed and recruited from the First Affiliated Hospital of Nanjing Medical University, the Jiangsu Institute of Cancer Research, Nanjing Drum Tower Hospital, the Nantong Tumor Hospital, and the Qidong Liver Cancer Institute in Jiangsu Province from January 2004 to April 2014. Those who had a history of cancer, metastasized cancer from other organs, radiotherapy, or chemotherapy were excluded from all the validation case groups. Information on demographic data was obtained from face-to-face interviews. Cancer-free controls were randomly selected from a cohort of more than 30,000 participants in a community-based screening program for noninfectious diseases in Jiangsu Province, China, which were frequency-matched to the cases based on age (5-year interval) and residential area (urban or rural). Hepatocellular carcinoma and colorectal cancer share the same cancer-free controls. Eventually, 7580 cases (3287 breast cancers, 1133 cervical cancers, 2044 hepatocellular carcinomas, and 1116 colorectal cancers) and 4874 cancer-free controls (2660 controls for breast cancer, 1098 controls for cervical cancer, and 1116 controls for both hepatocellular carcinoma and colorectal cancer) were included in this study. The blood samples of cases were collected after cancer confirmation, while control blood samples were prospectively collected after recruiting in the community-based cohort. For each participant, 5 ml of whole blood was obtained to extract genomic DNA.

### 2.3. *BRCA1/2* Pathogenic Variant Analysis, Nomenclature, and Screening

The exon regions of the *BRCA1* and *BRCA2* genes were amplified by 31 pair and 41 pair primers, respectively (Supplementary Tables 1 and 2), using the SureCycler 8800 (Agilent, Penang, Malaysia). Direct DNA Sanger sequencing was carried out by ABI PRISM BigDye Sequencing Kits and ABI 3730 Genetic Analyzer (Applied Biosystems, Foster City, CA, USA). The obtained sequences of *BRCA1* and *BRCA2* were aligned and analyzed using MEGA 5.0 software compared with GenBank accession numbers NM_007294.3 and NM_000059.3, respectively. We observed 48 variants in the discovery stage and selected candidate variants in validation stage with following criterions: (1) frameshift, nonsense, and missense variants; (2) minor allele frequency (MAF) less than 1% according to the Asian samples from 1000 Genomes Project [14]; and (3) the clinical classification identified by ENIGMA [15] was ascertained from BRCA exchange (https://brcaexchange.org/) ranging from Class 3 to Class 5. Then, we performed subsequent variant screening on the MassARRAY System (Agena Bioscience, San Diego, CA) among multicancers and the corresponding controls. For quality control, positive controls of candidate variants were used in each chip. Each recurrent variant identified from screening was doubly validated by Sanger sequencing.

### 2.4. Cell Culture

Human breast cancer cell lines MDA-MB-231 (triple negative, ER-/PR-/HER2-) and MCF-7 (luminal subtype, ER+/PR+/HER2-) were obtained from the American Type Culture Collection (ATCC, Manassas, VA, USA). The source and mycoplasma contamination of the MDA-MB-231 and MCF-7 cell lines were, respectively, evaluated by Cobioer Biosciences Co., Ltd. in August 2018 and by Shanghai Zhongqiao Technology Co., Ltd. in March 2017. Our MDA-MB-231 and MCF-7 cell lines were considered to be identical to the ATCC corresponding cell lines when the entered STR profiles yield 100% match to the ATCC STR database. No cross-contaminated cell lines or mycoplasma contamination was detected. Cells were incubated in DMEM (Gibco, Carlsbad, MA) and supplemented with 100 U/ml penicillin, 100 *μ*g/ml streptomycin, and 10% fetal bovine serum (Gibco, Carlsbad, MA) at 37°C in a humidified incubator (Thermo Forma, New York, USA) with 5% CO_2_.

### 2.5. Transfection

The pBABEpuro/wild-type *BRCA1* and pBABEpuro/del-T *BRCA1* plasmids were purchased from Addgene (Cambridge, MA 02139, USA). Cells were transfected with plasmids using Lipofectamine 2000 reagent (Invitrogen, Thermo Fisher Scientific, USA) with OPTI-MEM (Gibco, Carlsbad, MA) according to the manufacturer's instructions. GFP plasmid was also transfected in cells, and the transfection efficiency was detected by intensity of GFP expression using fluorescent microscopy in 24 hours posttransfection. After 48 hours of transfection, cells were harvested for the subsequent experiment.

### 2.6. RNA Extraction, Reverse Transcription, and Quantitative Real-Time PCR

Total RNA was extracted from cells using TRIzol Reagent (Invitrogen, Thermo Fisher Scientific, USA) under RNase-free conditions. Approximately 1,000 ng of RNA was used for the reverse transcription reaction with PrimeScript RT Master Mix (TaKaRa, Dalian, China). The purified cDNA was directly used as templates, and the quantitative real-time PCR (qRT-PCR) was performed by iTaq Universal SYBR Green Supermix (Bio-Rad #1725121) and QuantStudio 7 Flex Real-Time PCR System (Applied Biosystems, Foster City, CA, USA). The *BRCA1* primers were as follows: 5′-AAGAAAGAGGAACGGGCTTG-3′ (forward) and 5′-CCTCAAGGGCAGAAGAGTCA-3′ (reverse). The expression level was normalized by the internal control *GAPDH*, and the relative level of mRNA expression was calculated by equation 2–*Δ*CT (CT, cycle threshold; ΔCT = CT *BRCA*1–CT *GAPDH*).

### 2.7. Western Blot Assay

Total cell lysates were extracted using RIPA lysis buffer (Beyotime, China) and quantified using the BCA protein assay kit (Beyotime, China). Equal amounts of proteins were separated using 8% SDS-PAGE and transferred to a PVDF membrane (Millipore, Billerica, MA, USA). The membranes were blocked in 5% skim milk for one hour and then incubated overnight at 4°C with the following primary antibodies: HA-Tag (C29F4) rabbit mAb (1 : 1000 dilution; Cell Signaling Technology, USA), GAPDH (D16H11) XP rabbit mAb (1 : 1000 dilution; Cell Signaling Technology, USA). HA-tag (C29F4) rabbit mAb was used for detecting labeled *BRCA1* protein. Then, the membranes were incubated again with goat anti-rabbit IgG/goat anti-mouse IgG horseradish peroxidase- (HRP-) conjugated secondary antibodies (1 : 2000 dilution, Cell Signaling Technology, USA) for two hours at room temperature. Protein bands were visualized by using the ECL Plus western blotting detection reagents (Millipore, Billerica, MA, USA).

### 2.8. Cell Proliferation Assay

Cell proliferation was detected using the CCK-8 assay (Cell Counting Kit-8, Dojindo, Japan) and colony formation assay. A total of 3 × 10^3^ cells were seeded in 96-well plates at 0, 24, 48, 72, and 96 h, and 10 *μ*l of reaction solution was added to cells mixed with 100 *μ*l nonserum DMEM. After two hours of incubation, the absorbance of each plate was measured at 450 nm using a microplate reader (Molecular Devices, Sunnyvale, CA, USA). The values were obtained from six replicate wells for each condition and time point. All experiments were performed in triplicate. In the colony formation assay, 1 × 10^3^ transfected cells were seeded in 6-well plates and maintained for 10 days. The plates were photographed, and the number of visible colonies was counted. The assay was performed in triplicate.

### 2.9. Cell Migration Assay

Cell migration assays were performed using Costar Transwell plates (6.5 mm diameter inserts, 8.0 *μ*m pore size, polycarbonate membrane, Corning Sparks, MD). Then, 600 *μ*l DMEM containing 10% fetal bovine serum was added to the lower chamber, while 2 × 10^4^ cells in 300 *μ*l serum-free medium were added to the upper chamber. After 48 hours of incubation at 37°C, cells that had not migrated were removed from the upper surface of the membrane using a cotton swab. The remaining cells were then fixed with methanol for 15 minutes and stained with crystal violet solution for 20 minutes. We then used an optical microscope at a magnification of ×100 to visualize the stained cells in five random fields within each membrane. All assays were performed in triplicate, and the experiment was repeated three times.

### 2.10. Statistical Analysis

Variant frequencies, distribution differences among demographic characteristics, and selected variables between the cases and controls were analyzed using Student's *t* tests (for the continuous variables) or *χ*^2^ test (for the categorical variables). All statistical analyses were performed with R software (version 2.13.0; The R Foundation for Statistical Computing). *P* ≤ 0.05 in a two-sided test was considered statistically significant.

## 3. Results

### 3.1. Participant Characteristics

The baseline characteristics of multicancers and corresponding controls are summarized in Supplementary Table 3. Briefly, the distributions of age and age at natural menopause were similar between patients and controls. Earlier age at menarche and later age at first live birth were observed in breast cancer patients (*P* < 0.001). Among breast cancer cases with available ER, PR, and HER2 information both in discovery and validation stages, 1741 (54.12%) cases were ER positive, 1164 (36.18%) cases were PR positive, and 507 (15.76%) cases were HER2 positive. In addition, selected characteristics (gender and age) of three other malignancy cases and cancer-free controls were also described (Supplementary Table 3).

### 3.2. BRCA1 and BRCA2 Germline Variant Screening and Expanding Validation

**Figure 1:**
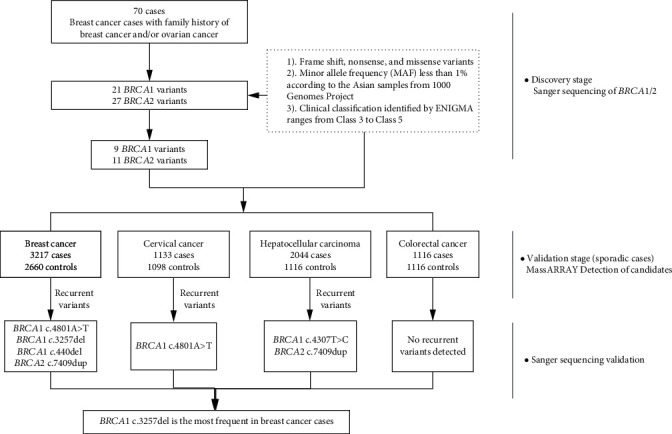
Flowchart of *BRCA1* and *BRCA2* germline variant selection.

**Figure 2:**
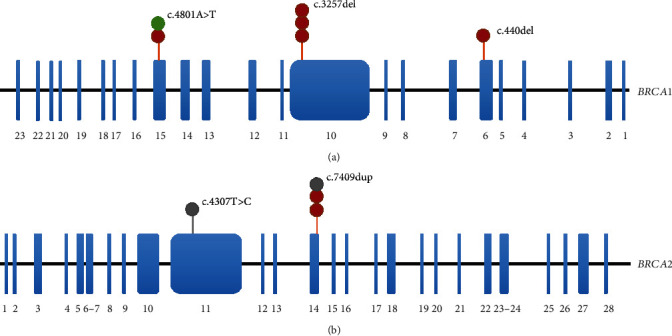
Recurrent pathogenic variants detected in the validation stage on exons of (a) *BRCA1* and (b) *BRCA2*. Exons are colored in blue; circles represent validated cases that are colored in red (breast cancer), green (cervical cancer), and gray (hepatocellular carcinoma).

The workflow of *BRCA1* and *BRCA2* germline variant detection and validation is shown in Figure 1. We successfully amplified and sequenced the exons of *BRCA1* and *BRCA2* in seventy breast cases with breast cancer and/or ovarian cancer family history, and a total of 48 variants were detected in discovery stage (Supplementary Table 4). According to our selection criterion of variants, 20 potentially pathogenic variants containing 9 variants in *BRCA1* and 11 variants in *BRCA2* were detected in at least one case from 70 familial breast cancer cases (Table 1). Two variants (*BRCA1* c.4460A>G and c.824G>A) were recurrent in discovery stage in two patients with breast cancer and/or ovarian cancer family history. Four novel VUS in *BRCA1* (c.440del) and *BRCA2* (c.4207A>G, c.7093C>A, and c.7149T>A) were first reported in our study. To further evaluate whether *BRCA1/2* potentially pathogenic variants could recur in larger sporadic breast cancer cases and/or other cancer types, we designed our own panel with the 20 pathogenic variants above. The multistage of validation was implemented in breast cancer, cervical cancer, hepatocellular carcinoma, and colorectal cancer. Counts of potentially pathogenic variants are listed in Table 2 and Supplementary Table 5. Five recurrent variants in sporadic cases (*BRCA1* c.4801A>T, *BRCA1* c.3257del, *BRCA1* c.440del, *BRCA2* c.4307T>C, and *BRCA2* c.7409dup) were identified and three of them were labeled Class 5 by ENIGMA. The distributions of these variants on *BRCA1* and *BRCA2* are described in Figure 2. It is noteworthy that *BRCA1* c.3257del was recurrent in three unrelated cases among sporadic breast cancer cases. This deletion results in a termination codon, probability leading to shortened peptide chains terminated at the 1086 protein position. Although *BRCA1* c.3257del was recorded in the ClinVar database (Variation ID: 252873, http://www.ncbi.nlm.nih.gov/clinvar/), there was no explicit research reporting this finding.

Interestingly, we also found that the *BRCA2* c.7409dup was harbored in two unrelated sporadic breast cancer patients and one hepatocellular carcinoma patient. Previous studies have reported that this pathogenic variant exists in familial breast and/or ovarian cancer from Hong Kong and Shanghai populations but without recurrent patients [16, 17]. *BRCA1* c.4801A>T was recorded in Breast Cancer Information Core (BIC) in Chinese [18] and appeared to be recurrent in one breast cancer and one cervical cancer patient in the present study (sample ID: 71, 78, Table 3). Moreover, *BRCA2* c.4307T>C occurred in a hepatocellular carcinoma patient with a family history of breast cancer. However, no candidate variants were detected in colorectal cancer cases. The sequences of all variants are listed in Supplementary Figure 1.

### 3.3. Association between BRCA1/2 Variants and Clinical Characteristics of Breast Cancer, Cervical Cancer, Hepatocellular Carcinoma, and Colorectal Cancer

Combined with the discovery and validation stages of breast cancer, 31 cases carried 20 *BRCA1/2* variants, with one case (sample ID: 26) harboring two variants. We summarized the characteristics of all samples with variants in Table 3. The mean age at diagnosis for these positive cases was 49.23 (ranging from 27 to 68 years old). Our data showed that all four patients who carried the *BRCA1* c.3257del had ER/PR-negative status. This phenomenon suggested that *BRCA1* c.3257del might contribute to breast cancer development with a specific ER/PR status. Intriguingly, more than 30% (8/25) of patients with available ER/PR status were observed as triple-negative breast cancer cases, which was apparently higher than its epidemiological distribution of approximately 15% [19]. Among these triple-negative cases, case 26 harbored *BRCA1*c.5133del and *BRCA2* c.7093C>A diagnosed at only 30 years old.

### 3.4. BRCA1 c.3257del Reduced mRNA and Protein Expressions of BRCA1 in MDA-MB-231

**Figure 3:**
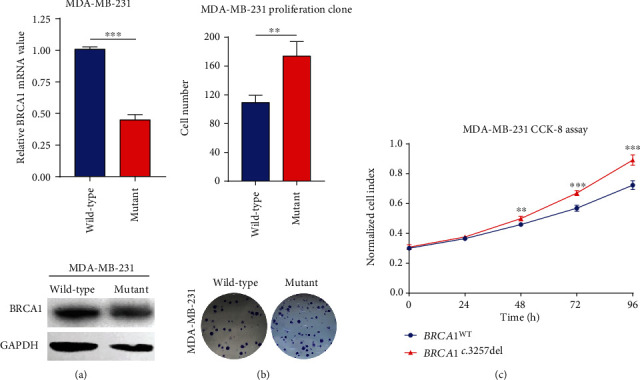
(a) The *BRCA1* mRNA level of MDA-MB-231 transfected with *BRCA1*^c.3257del^ plasmids was significantly reduced (*P* < 0.05) compared to those transfected with wild-type *BRCA1* plasmids. Western blot analysis showed that the *BRCA1* protein level translated by *BRCA1*^c.3257del^-overexpressed cells was lower than *BRCA1*^WT^ in MDA-MB-231 cells. (b) The colony formation efficiency of MDA-MB-231 cells transfected with *BRCA1*^c.3257del^ was significantly increased (*P* < 0.05). (c) Cell proliferation of *BRCA1*^c.3257del^ (red) and *BRCA1*^WT^ (blue) was measured using the CCK-8 assay. Increased proliferation of *BRCA1*^c.3257del^-overexpressing MDA-MB-231 cells was detected in vitro (*P* < 0.05).

Since *BRCA1* c.3257del is classified as a frameshift variant that theoretically leads to the termination codon and most frequently leads to ER/PR-negative status in breast cancer, we turned our investigation to the alterations of *BRCA1* mRNA and protein expressions in breast cancer cell lines. MCF-7 cells and MDA-MB-231 cells were transfected with *BRCA1*^WT^ and *BRCA1*^c.3257del^ plasmids, respectively, and significantly reduced mRNA and protein expression was observed in cells transfected with *BRCA1*^c.3257del^ compared with wild-type cells (*P* < 0.001, Figure 3(a)). However, a similar phenomenon was not observed in MCF-7 cell lines, which showed ER- and PR-positive characteristics (Supplementary Figure 2).

### 3.5. BRCA1 c.3257del Increased the Proliferation of Triple-Negative Breast Cancer Cells

To identify the functions of the most recurrent variant *BRCA1*^c.3257del^ on breast cancer cells, we performed CCK-8 assays, colony formation assays, and transwell assays. As expected, significantly increased proliferation was observed in MDA-MB-231 cells transfected with *BRCA1*^c.3257del^ compared with wild-type plasmids using CCK-8 and colony formation assays (*P* < 0.001, Figures 3(b) and 3(c)). No significant change in migration was exhibited in MDA-MB-231 (Supplementary Figure 2D). However, the proliferation and migration of MCF-7 cells revealed no difference after transfection with *BRCA1*^c.3257del^ and wild-type plasmids (Supplementary Figure 2).

## 4. Discussion

We performed a detection of *BRCA1/2* exons in a total of 7580 cases and 4874 controls spanning four cancers. A total of 20 pathogenic variants or VUS were discovered in 70 breast cancer cases with BC and/or OC family history, with five recurrently pathogenic variants identified in validation stages (Table 2), including cervical cancer and hepatocellular carcinoma. Among these variants, four recurrent variants (*BRCA1* c.3257del, *BRCA1* c.4801A>T, *BRCA1* c.440del, and *BRCA2* c.7409dup) might be considered as the candidate locus in breast cancer screening.

It is well known that the tumor suppressor genes *BRCA1* and *BRCA2* play a critical role in homologous recombination, which is considered to be the major mechanism for genome integrity in the process of cell proliferation [20]. As the functions of *BRCA1/2* have been studied thoroughly, certain deleterious mutations on *BRCA1/2* were verified as predisposing with familial breast and ovarian cancer [21–23]. For example, *BRCA1* c.68_69del, *BRCA1* c.5266dup, and *BRCA2* c.5946del were three founder deleterious mutations among the European population [24] and could be applied in high-risk population screening and carriers can acquire appropriate genetic counseling. Rebbeck et al. studied 1650 unique *BRCA1* and 1731 unique *BRCA2* deleterious mutations from 29,700 families worldwide and observed distinct variation in mutation type or frequency by geographical region and race/ethnicity [25]. Considering the heterogeneity of countries and ethnicity, the effects of *BRCA1/2* pathogenic mutations in Westerners were extraordinarily weaker among the Chinese population [8].

To date, several studies have reported the *BRCA1/2* pathogenic variants from breast or ovarian cancer in Chinese populations with diverse results. A meta-analysis collected 94 publications with 2128 *BRCA1/2* variant records which showed several high-frequency variants but no potential founder variants were identified [26], for example, the two highest recurrent variants c.5470_5477delATTGGGCA [27–29] in *BRCA1* and c.3109C>T in *BRCA2* [25, 30]. Recently, You et al. identified five recurrent variants in 172 Chinese women with epithelial ovarian cancer, including three pathogenic mutations (*BRCA1* c.5470_5477delATTGGGCA and c.66dup and *BRCA2* c.1963delC) and two VUS missense mutations (*BRCA2* c.1568A>G and c.6325G>A) [29]. However, these potentially pathogenic mutations above were not validated in our current study. It is possible that the prevalence of recurrent pathogenic variants is various according to the geographical origin of the studied patients or probably that tumorigenesis might be triggered by diversely deleterious mutations partly. Some formerly reported benign loci were also observed in our discovery stage. For example, a Korean study revealed that *BRCA1* c.2566T>C showed a genotype frequency in cases greater than 2% compared with the control group [31]. In addition, *BRCA2* c.8187G>T repeated in three cases of our discovery stage was also associated with moderate/low risk of breast cancer in a total of 164 cases and 128 controls from a combined study containing nine studies of Asian ancestry [32]. Due to the relatively high frequency in 1000 Genomes Project in Asian population, these two variants were excluded in our validation stage.

In addition to the known founder deleterious mutations, recurrent variants of relatively high frequency in specific race/ethnicity may be used in targeted (panel) genotyping for genetic testing in specific populations more efficiently [25]. In our multistage study of breast cancer, 21 of 70 cases (40.0%) in the discovery stage carried 20 pathogenic variants or VUS in *BRCA1/2* in Chinese women. Subsequent validation with 3217 sporadic breast cancers and 2660 healthy controls found seven breast cancer patients carried five recurrent variants from these *BRCA1/2* pathogenic variants mentioned above. The most frequent variant *BRCA1* c.3257del was detected in four unrelated patients with ER-/PR- status, and two patients were undertaking triple-negative breast cancer. According to our results, we speculated that the *BRCA1* c.3257del might weaken the function of the tumor suppressor gene *BRCA1*. Interestingly, we noticed that *BRCA1* c.3257del downregulated *BRCA1* mRNA/protein expression and promoted proliferation of triple-negative MDA-MB-231 cell line. These observations did not occur in the MCF-7 cell line (ER+/PR+), suggesting that *BRCA1* c.3257del might influence transcriptional and posttranscriptional regulations to encumber the *BRCA1* functions, especially in triple-negative breast cancer. Together with the compelling proliferative effects of *BRCA1* c.3257del revealed in the current study, we considered this pathogenic variant might especially contribute to the development of triple-negative breast cancer.

Although *BRCA1* and *BRCA2* as double-stranded DNA repair genes preferentially promote tumorigenesis of breast and ovarian epithelial cells, supportive evidence still exists that shows that *BRCA1/2* might play a role in the progression or prognosis of other cancer types [6]. Using TCGA publicly available expression profiles of cancer patients, we found that *BRCA1* and *BRCA2* were widely expressed in multiple tumors compared with normal tissues (Supplementary Figure 3), including breast invasive carcinoma (BRCA), liver hepatocellular carcinoma (LIHC), and rectum adenocarcinoma (READ). Since divergent expressions of suppressor genes or oncogenes usually carry pathogenic or likely pathogenic variants [13], the variants located in specific genes might be responsible for the abnormal expressions and implied in tumorigenesis and progression. Some independent evidence provides differential expression of *BRCA1/2* in other tumors. Ferroudj et al. found that *BRCA1* and *BRCA2* expression levels display a gradual increase in parallel in tumor progression from early to advanced stages of hepatocellular carcinoma (HCC) by utilizing human hepatocellular carcinoma microarrays in the Gene Expression Omnibus database [33]. In addition, a previous study demonstrated that the expression/protein levels of *BRCA1* were increased in noncomplete response (NCR) compared to complete response (CR) cervical tumors [34]. In the present study, we corroborated the potential impacts of *BRCA1*/*2* pathogenic variants or VUS in other cancers and detected two hepatocellular carcinoma patients carrying *BRCA2* variants (c.7409dup and c.4307T>C) and one cervical cancer patient harboring *BRCA1* c.4801A>T. Overall, mutant *BRCA1/2* is an indispensable founding event for some tumors, but appears to be biologically neutral or incidental in others [35]. Although we do not have substantial evidence that these recurrent *BRCA1/2* variants are associated with cervical cancer and hepatocellular carcinoma, it still expanded the consideration of the potential role of *BRCA1/2* for other cancer types.

## 5. Conclusions

We reported five recurrent variants of *BRCA1/2* in multistage studies with 7580 cases and 4874 controls. Our findings also provided suggestive evidence that *BRCA1* c.3257del could cause a decline in *BRCA1* mRNA/protein expression and promote cell proliferation, especially in triple-negative breast cancer. However, there remains a limitation. First, the specificity among different cancers might weaken the persuasion of pathogenic variants validated in breast cancer and only focusing on *BRCA1/2* exons might miss some potential deleterious variants. Since point variants at exon-intron boundary sequences can cause improper exon or intron recognition and might result in the formation of an aberrant transcript of the mutated gene, more comprehensive detection covering whole *BRCA1/2* exon and exon-intron boundaries would be taken in further expanding validation. Second, tumorigenesis was a complicated process accompanied with multievent hits, such as germline variants cooccurred with somatic variants (copy-number variations, eccDNA, allelic imbalance, etc.), environmental stimulations, and even virus infections. The insufficiency of HPV and HBV information in our study might underestimate virus infections, especially in HPV-related cervical cancer and HBV-related hepatocellular carcinoma. As a result, the current data were focused on the germline mutations which do not necessarily represent the entire real pathogenic events. Moreover, the intensive mechanism of *BRCA1* c.3257del implicated in TNBC development and progress was not deep enough and needs further exploration. More validations are warranted to verify our results. Overall, the present study not only enriched the pathogenic variant spectrum of *BRCA1/2* but also suggested that a high-risk Chinese population of breast cancer might benefit from genetic screening using these recurrent loci.

## Figures and Tables

**Table 1:** *BRCA1/2* germline variants in Chinese patients with breast cancer who had a family history of breast cancer and/or ovarian cancer (*N* = 70).

Gene	Transcript	Exons	Location (GRCh37)	Counts	Variant type	Nucleotide change	Effect on amino acids	References	Classification of ENIGMA^a^
*BRCA1*	NM_007294.3	ENSE00003591784	chr17:41215910-41215910	1/70	Frameshift	c.5133del	p.(Lys1711AsnfsTer3)	BIC	Class 3
*BRCA1*		ENSE00003497952	chr17:41223130-41223130	1/70	Nonsense	c.4801A>T	p.(Lys1601Ter)	BIC	Class 5
*BRCA1*		ENSE00003791246	chr17:41228529-41228529	2/70	Missense	c.4460A>G	p.(Lys1487Arg)	BIC	Class 3
*BRCA1*		ENSE00003522602	chr17:41244100-41244100	1/70	Missense	c.3448C>T	p.(Pro1150Ser)	BIC	Class 3
*BRCA1*		ENSE00003522602	chr17:41244291-41244291	1/70	Nonsense	c.3257del	p.(Leu1086Ter)	BIC	Class 5
*BRCA1*		ENSE00003522602	chr17:41245587-41245587	1/70	Frameshift	c.1961del	p.(Lys654SerfsTer47)	BIC	Class 5
*BRCA1*		ENSE00003522602	chr17:41245847-41245848	1/70	Frameshift	c.1700dup	p.(Asn567LysfsTer3)	BIC	Class 5
*BRCA1*		ENSE00003522602	chr17:41246724-41246724	2/70	Missense	c.824G>A	p.(Gly275Asp)	BIC	Class 3
*BRCA1*		ENSE00003541068	chr17:41256140-41256140	1/70	Frameshift	c.440del	p.(Leu147CysfsTer16)	Novel	Class 3

*BRCA2*	NM_000059.3	ENSE00003659301	chr13:32899249-32899249	1/70	Missense	c.353G>A	p.(Arg118His)	BIC	Class 3
*BRCA2*		ENSE00000939168	chr13:32910452-32910452	1/70	Missense	c.1960G>A	p.(Glu654Lys)	BIC	Class 3
*BRCA2*		ENSE00000939168	chr13:32910963-32910963	1/70	Nonsense	c.2471T>G	p.(Leu824∗)	BIC	Class 5
*BRCA2*		ENSE00000939168	chr13:32911757-32911757	1/70	Nonsense	c.3265C>T	p.(Gln1089∗)	BIC	Class 5
*BRCA2*		ENSE00000939168	chr13:32912699-32912699	1/70	Missense	c.4207A>G	p.(Thr1403Ala)	Novel	Class 3
*BRCA2*		ENSE00000939168	chr13:32912799-32912799	1/70	Missense	c.4307T>C	p.(Ile1436Thr)	BIC	Class 3
*BRCA2*		ENSE00000939168	chr13:32914173-32914174	1/70	Nonsense	c.5681dup	p.(Tyr1894∗)	BIC	Class 5
*BRCA2*		ENSE00000939173	chr13:32929083-32929083	1/70	Missense	c.7093C>A	p.(His2365Asn)	Novel	Class 3
*BRCA2*		ENSE00000939173	chr13:32929140-32929140	1/70	Nonsense	c.7149T>A	p.(Tyr2383∗)	Novel	Class 3
*BRCA2*		ENSE00000939173	chr13:32929399-32929400	1/70	Frameshift	c.7409dup	p.(Thr2471Hisfs∗4)	BIC	Class 5
*BRCA2*		ENSE00000939174	chr13:32930651-32930651	1/70	Missense	c.7522G>A	p.(Gly2508Ser)	BIC	Class 3

^a^The ENIGMA classification was ascertained from BRCA exchange.

**Table 2:** Five recurrent variants of *BRCA1/2* in validation stages.

Gene	Nucleotide change	Validation stage							
		Breast cancer		Cervical cancer		Hepatocellular carcinoma		Colorectal cancer	
		Cases (*N* = 3217)	Controls (*N* = 2660)	Cases (*N* = 1133)	Controls (*N* = 1098)	Cases (*N* = 2044)	Controls^a^ (*N* = 1116)	Cases (*N* = 1116)	Controls^a^ (*N* = 1116)
*BRCA1*	c.4801A>T	1/3217	0/2660	1/1133	0/1098	0/2044	0/1116	0/1116	0/1116
*BRCA1*	c.3257del	3/3217	0/2660	0/1133	0/1098	0/2044	0/1116	0/1116	0/1116
*BRCA1*	c.440del	1/3217	0/2660	0/1133	0/1098	0/2044	0/1116	0/1116	0/1116
*BRCA2*	c.4307T>C	0/3217	0/2660	0/1133	0/1098	1/2044	0/1116	0/1116	0/1116
*BRCA2*	c.7409dup	2/3217	0/2660	0/1133	0/1098	1/2044	0/1116	0/1116	0/1116

^a^Hepatocellular carcinoma and colorectal cancer shared the cancer-free control samples.

**Table 3:** Clinical characteristics of cases harboring *BRCA1/2* germline variants.

Gene	Nucleotide change	Counts	Sample ID	Cancer type^a^	Stage	Age	Family history^a^	ER	PR	HER2
*BRCA1*	c.5133del	1	26	BC	Discovery	30	BC	—	—	—
	c.4801A>T	3	5	BC	Discovery	34	BC	NA	NA	NA
			71	BC	Validation	46		—	—	—
			78	CC	Validation	44		NA	NA	NA
	c.4460A>G	2	21	BC	Discovery	50	BC	+	+	NA
			23	BC	Discovery	48	BC	—	—	—
	c.3448C>T	1	12	BC	Discovery	45	BC	+	+	+
	c.1961del	1	67	BC	Discovery	54	OC	—	+	—
	c.1700dup	1	40	BC	Discovery	53	BC	—	—	+
	c.824G>A	2	4	BC	Discovery	46	BC	—	—	+
			70	BC	Discovery	27	BC	NA	NA	NA
	c.3257del	4	62	BC	Discovery	37	OC	—	—	—
			72	BC	Validation	58		—	—	+
			73	BC	Validation	62		—	—	+
			74	BC	Validation	37		—	—	—
	c.440del	2	50	BC	Discovery	48	BC	—	—	—
			75	BC	Validation	60		+	+	—

*BRCA2*	c.4307T>C	2	64	BC	Discovery	60	OC	—	—	+
			79	HCC	Validation	58	BC	NA	NA	NA
	c.7409dup	4	18	BC	Discovery	50	BC	NA	NA	NA
			76	BC	Validation	55		—	—	NA
			77	BC	Validation	46		+	+	NA
			80	HCC	Validation	68		NA	NA	NA
	c.7093C>A	1	26	BC	Discovery	30	BC	—	—	—
	c.3265C>T	1	57	BC	Discovery	50	BC	+	+	—
	c.4207A>G	1	35	BC	Discovery	44	BC	—	—	—
	c.353G>A	1	37	BC	Discovery	33	BC	+	+	+
	c.1960G>A	1	25	BC	Discovery	60	BC	NA	NA	NA
	c.7149T>A	1	69	BC	Discovery	54	BC	—	—	+
	c.7522G>A	1	3	BC	Discovery	53	BC	—	+	+
	c.2471T>G	1	10	BC	Discovery	41	BC	—	—	+
	c.5681dup	1	61	BC	Discovery	38	BC	+	+	+

^a^BC: breast cancer; CC: cervical cancer; HCC: hepatocellular carcinoma; OC: ovarian cancer.

## Data Availability

The data used to support the findings of this study are available from the corresponding author upon request.

## Abbreviations

BC: Breast cancer
BCLC: Breast Cancer Linkage Consortium
BIC: Breast Cancer Information Core
BRCA: Breast invasive carcinoma
CC: Cervical cancer
CR: Complete response
eccDNA: Extrachromosomal circular DNA
HCC: Hepatocellular carcinoma
LIHC: Liver hepatocellular carcinoma
NCR: Noncomplete response
OC: Ovarian cancer
qRT-PCR: Quantitative real-time PCR
READ: Rectum adenocarcinoma
TCGA: The Cancer Genome Atlas
VUS: Variants of uncertain significance.

## Supplementary Materials

Supplementary Materials Supplementary Figure 1: sequence results of 20 detected variants in 31 cases in BRCA1 and BRCA2. Supplementary Figure 2: (A) the mRNA level of MCF-7 transfected with different plasmids showed no significant difference in different groups (*P* > 0.05). Western blot analysis showed that the BRCA1 proteins translated by BRCA1 c.3257del were not truncated in MCF-7 cells. (B) The colony formation efficiency of MCF-7 transfected with BRCA1 c.3257del was not increased (*P* > 0.05). (C) BRCA1 c.3257del-overexpressing MCF-7 cell proliferation efficiency was not increased as measured by the CCK-8 assay in vitro (*P* > 0.05). (D) Both MDA-MB-231 and MCF-7 cell migration was measured by transwell assay, and no significant difference in cell migration was detected (*P* > 0.05). Supplementary Figure 3: BRCA1 and BRCA2 expression data from TCGA in multiple tumors compared with paired normal tissues. Supplementary Table 1: information of primers for BRCA1. Supplementary Table 2: information of primers for BRCA2. Supplementary Table 3: distributions of selected variables in multicancer cases and cancer-free controls. Supplementary Table 4: 48 variants obtained from BRCA1/2 scanning in 70 familial breast cancer cases. Supplementary Table 5: no recurrent BRCA1/2 germline variants in validation stages.
