# Supplementary material for: Identification of Recurrent Variants in BRCA1 and BRCA2 across Multiple Cancers in the Chinese Population
Source: Biomed Res Int. 2020 Aug 15;2020:6739823. doi: 10.1155/2020/6739823 (PMC7448236; doi:10.1155/2020/6739823)
Supplement: Supplementary Materials — Supplementary Figure 1: sequence results of 20 detected variants in 31 cases in BRCA1 and BRCA2. Supplementary Figure 2: (A) the mRNA level of MCF-7 transfected with different plasmids showed no significant difference in different groups (P > 0.05). Western blot analysis showed that the BRCA1 proteins translated by BRCA1 c.3257del were not truncated in MCF-7 cells. (B) The colony formation efficiency of MCF-7 transfected with BRCA1 c.3257del was not increased (P > 0.05). (C) BRCA1 c.3257del-overexpressing MCF-7 cell proliferation efficiency was not increased as measured by the CCK-8 assay in vitro (P > 0.05). (D) Both MDA-MB-231 and MCF-7 cell migration was measured by transwell assay, and no significant difference in cell migration was detected (P > 0.05). Supplementary Figure 3: BRCA1 and BRCA2 expression data from TCGA in multiple tumors compared with paired normal tissues. Supplementary Table 1: information of primers for BRCA1. Supplementary Table 2: information of primers for BRCA2. Supplementary Table 3: distributions of selected variables in multicancer cases and cancer-free controls. Supplementary Table 4: 48 variants obtained from BRCA1/2 scanning in 70 familial breast cancer cases. Supplementary Table 5: no recurrent BRCA1/2 germline variants in validation stages. [file 6739823.f1.pdf]

Supplementary table 1. Information of primers for *BRCA1*

Exons of *BRCA1*	Primers
ENSE00001871077	F:AAGGGGTTGGCAGCAATATG<br>R:AGGCAGAGTGGATGGAGAAC
ENSE00003559512	F:TTGAACGAACTTGAGGCCTT<br>R:TATGTCAGTCGGGTGTGGTG
ENSE00003510592	F:GGCTCTTAAGGGCAGTTGTG<br>R:GCGATACAGCCCTACTTTACA
ENSE00003541068	F:CACTGCCATCACACGGTTTA<br>R:GCTAAGACACCAACAATGTAAGT
ENSE00003531836	F:TGTTGGTGTCTTAGCTTTAGTGA<br>R:CAATTTGGGGAGCCGAGGT
ENSE00003513709	F:TCCCAGCCCCAGACATTTTA<br>R:CCAAAGCTGCCTACCACAAA
ENSE00003642045	F:TGCCACAGTAGATGCTCAGT<br>R:GCACATACATCCCTGAACCT
ENSE00003587679	F:GCCCAGCAACCATTTCATTTC<br>R:GGTTGTAAAGGTCCCAAATGG
ENSE00003522602	1F:TGGTTGATTTCCACCTCCAAG<br>1R:ACACAGGGGATCAGCATTCA<br>2F:ACTCATGCCAGCTCATTACAG<br>2R:CATGGCTTAAGTTGGGGAGG<br>3F:GCCATGCTCAGAGAATCCTAG<br>3R:ACTGCTGCTTATAGGTTCAGC<br>4F:TGGCAGTTCAAAAGACTCCTG<br>4R:CTTCTTGGCTCCAGTTGCAG<br>5F:ACGAAAGCTGAACCTATAAGCAG<br>5R:AGTGCCATAATCAGTACCAGGT<br>6F:GCCAAGAAGAGTAACAAGCCA<br>6R:TGTTTCTTTAAGGACCCAGAGTG<br>7F:CACAGTCGGGAAACAAGCAT<br>7R:CGGCTAATTGTGCTCACTGT<br>8F:GGCAACGAAACTGGACTCAT<br>8R:TGTGTATGGGTGAAAGGGCT<br>9F:TGGGAAGTAGTCATGCATCTCA<br>9R:TGTAAAATGTGCTCCCCAAAAG
ENSE00003547126	F:AAAGCAAATCCAGGTGTCCC<br>R:GCAGTGTATGTGTAGGCTGTG
ENSE00003527960	F:AGGTGATTTCAATTCCTGTGCT<br>R:ACATGAATGACTGCCTTGGG
ENSE00003791246	F:GTCTGTTGCATTGCTTGTGT<br>R:AAGGGCTGCCTTGAAGAAGT
ENSE00003537850	F:GACTTCTAGGCTGTCTTGCG

	R:ACACCAAGACTCCCTCATCC
ENSE00003497952	1F:ACCAACACTGTATTCATGTACCC<br>1R:AATTCTGGCTTCTCCCTGCT<br>2F:GCCTCTTCTCTGATGACCCT<br>2R:TGGCAGTACATTTGGAACCAG
ENSE00003492626	F:TTTAAATAGTTCCAGGACAC<br>R:CTCCCAAAGTGCTGCGATTA
ENSE00003591784	F:GCCTCCCCTAGACTTCCAAAT<br>R:GGTAACTCAGACTCAGCATCAG
ENSE00003672792	F:CCTCTGTCATTCTTCCTGTGC<br>R:GTGGTGCATTGATGGAAGGA
ENSE00003458468	F:GCCTTAAATATGACGTGTCTGCT<br>R:AAAATGAAGCGGCCCATCTC
ENSE00003477922	F:CTGGACATTGGACTGCTTGT<br>R:GCTGTTTTGTTTGGAGAGTGG
ENSE00003628864	F:TCCTTTGGAGCAGAGAGACAG<br>R:GCAGTTCTCAAATCCTTACCCA
ENSE00003687053	F:GGTGGTGGTACGTGTCTGTA<br>R:AAATGTGCCAAGAACTGTGC
ENSE00001814242	F:ACTCATACAACCAGGACCCT<br>R:ACCCTTGCATAGCCAGAAGT

Supplementary table 2. Information of primers for *BRCA2*

Exons of *BRCA2*	Primers
ENSE00001484009	F:AGCGCTTCTGAGTTTTACCTC
	R:AGCAACACTGTGACGTACTG
ENSE00003666217	F:GGGGTTTCAACGTGTTAGCA
	R:GCCCAGCATGACACAATTAATG
ENSE00003659301	F:CCCTATACATTCTCATTCCCAGT
	R:TGTAAGATCTTCTACCAGGCTCT
ENSE00003739878	F:AATACACGGTTTCCAGCAGC
ENSE00003747332	R:GATCCACCTCAGCTCCTAGAC
ENSE00003749714	F:GCGTTATACCTTTGCCCTGA
	R:ATGCTTGACACCACTGGACT
ENSE00003714754	F:ACCAAGCCATATCTTACCACCT
	R:ACAGAGAGACAGCAGAGTTTCA
ENSE00003731761	F:TGGACCTAGGTTGATTGCAG
	R:TGAGGCAGGATCGCTTGA
ENSE00000939167	1F:AACAGGAGAAGGGGTGACTG
	1R:GGTAGGCTAGAAATACGTGGC
	2F:GGCCTGTGAATGGTCTCAAC
	2R:GCTTCAAACTGGGCTGAACA
	3F:TGCTCACAGAAGGAGGACTC
	3R:ACCATGTTTGAGTGACCTGAT
ENSE00000939168	1F:CCTGAGTAACTGGCACCACA
	1R:TGTTGTACTGGGTGACATGC
	2F:TCTGAAGAACCAACTTTGTCCT
	2R:GGCAACAGCTCAACGTTTT
	3F:CAGCACTCTTATTTTAACTC
	3R:CATTTTGCTCTTCTTAATGT
	4F:ACAAGCAACCCAAGTGTCAA
	4R:TCAACAGGCCAGCAAACTTC
	5F:ACCCCTCAGATGTTATTTTCCA
	5R:TGAGAATTTCTACTGGCAGCAG
	6F:AAGTGGGGTTTAGGGGCTTT
	6R:TCCCACTTGCAGTCTGAAAA
	7F:GGCCAGTTTATGAAGGAGGG
	7R:TGGCACCACAGTCTCAATAGA
	8F:GGTTTTCATACAGCTAGCGGG
	8R:GCTGTTAGACATGCTACTGTTAC
	9F:TCAGTCCCCTTATTCAGTCATTG
	9R:CTGAATCATCCAATGCCTCGT
	10F:TGTGACTAGCTCTTCACCCTG
	10R:TGTTCTGGAGTACGTATAGCAGT
	11F:CAGCAAGTGGAAAATCTGTCCA
	11R:AGCCTGTTCTTTTCCCAAAAC

	12F:TGATAAGAGAAACCCAGAGCACT<br>12R:TCCTCATTTTCGGGACATGTAA<br>13F:TGGGAAAAGAACAGGCTTCAC<br>13R:GCCTCCCGGAAACCAACT
ENSE00000939169	F:GCTTATCTGTGGTATCTGGTAGC<br>R:ACTTTGAGAGGCAGGTGGAT
ENSE00000939171	F:TGAGCATCTGTTACATTCACTGA<br>R:TTAACTGATTCGGAGCAATTTCC
ENSE00000939173	F:GCAAATGAGGGTCTGCAACA<br>R:AGGGCTTTAAAATTACCACCACC
ENSE00000939174	F:CCACACCTGGCTACTTTTGT<br>R:GCACAGATCACTTTAGCAGGA
ENSE00000939175	F:GGGGTGGAAAAGGTACAGCA<br>R:TAAACCCCAGGACAAACAGC
ENSE00001394102	F:GGGGTCTCACTATGTTGCCT<br>R:AGGGATGACAGGAGAACAGC
ENSE00000939177	1F:CTTGCTTCCCTCTTTCCCCA<br>1R:ACCAACTGTCAGTCTGCCAT<br>2F:GGGATGACACAGCTGCAAAA<br>2R:GAGAACAAGAGGGCAGCAAG
ENSE00000939178	F:TCTCTCTAGTTGCCTTTGTCCA<br>R:TGGCTCAGCTACTTGAGAGG
ENSE00000939180	F:TCCACTAATCTCAGCCTCCC<br>R:AGGTCCTAGTTCCAGGCATA
ENSE00003461148	F:CTTTAAATCTCCCTTCTTTG<br>R:CTGGCACATCACTGAAAATC
ENSE00000939183	F:ACCACACCCTTAAGATGAGCT<br>R:CTTTGTGGGCATTAGTAGTGGA
ENSE00000939185	F:CCACTACTAATGCCCACAAAGA<br>R:CCACCTCAGAACAAGATGGC
ENSE00000939187	F:CACTTCTTCCATTGCATCTTTCT<br>R:AGGATGTAACTTGTTACCCTTGA
ENSE00000939189	F:TGGAAAACCTGAGCTTTCGC<br>R:TCCTGAGGTTCATGGGCAAT
ENSE00003560258	F:TCTCTGTTCCCCTCTCCCTAT<br>R:ACAGGAGCCACATAACAACC
ENSE00001184789	1F:GGGAGGGAGACTGTGTGTAA<br>1R:CTGAACTGGTGGGAGCAGT<br>2F:GAGTTGTGGCACCAAATACGA<br>2R:CCGATACACAAACGCTGAGG

Supplementary Table 3. Distributions of selected variables in multi-cancer cases and cancer-free controls

Variables	Discovery Stage	Validation Stage		P value
	Breast Cancer	Breast Cancer		
	Case (N = 70)	Case (N = 3217)	Control (N = 2660)	
**Gender**				
Male	0(0%)	0(0%)	0(0%)	
Female	70(100%)	3217(100%)	2660(100%)	
**Age, years (mean ± s.d.)**[a]	50.04±9.45	50.19±11.12	50.42±11.87	0.454
**Age at menarche, years (mean ± s.d.)**[b]	14.57±1.55	14.74±1.86	15.86±2.08	**<0.001**
**Age at natural menopause, years (mean ± s.d.)**[d]	49.05±4.75	49.05±4.41	48.94±4.37	0.543
**Age at first live birth, years (mean ± s.d.)**[c]	26.58±3.29	25.58±3.28	24.38±3.30	**<0.001**
**Menopausal status** [e]				**<0.001**
Premenopausal	30(42.86%)	1589(49.39%)	1359(51.10%)	
Natural menopausal	34(48.57%)	1341(41.68%)	1260(47.37%)	
Unnatural menopausal	6(8.57%)	150(4.66%)	18(0.7%)	
**Estrogen receptor (ER)** [f]				
Positive	23(32.86%)	1718(53.40%)		
Negative	30(42.86%)	1134(35.25%)		
**Progesterone receptor (PR)** [f]				
Positive	23(32.86%)	1594(49.55%)		
Negative	27(38.57%)	1247(38.76%)		
**Human epidermal growth factor receptor-2 (HER2)** [f]				
Positive	9(12.86%)	280(8.70%)		
Negative	34(48.57%)	1109(34.47%)		

(Continued) Supplementary Table 3. Distributions of selected variables in multi-cancer cases and cancer-free controls

Variables	Validation Stage								
	Cervical Cancer		*P* value	Hepatocellular Carcinoma		*P* value	Colorectal Cancer		*P* value
	Case(N=1133)	Controls(N=1098)		Case(N=2044)	Controls [g](n=1116)		Cases [h](N=1116)	Controls [g](N=1116)	
**Gender** [a]									
Male	0(0%)	0(0%)		1731(84.69%)	918(82.26%)		662(59.32%)	918(82.26%)	
Female	1133(100%)	1098(100%)		313(15.31%)	198(17.74%)		433(38.80%)	198(17.74%)	
**Age, years (mean ± s.d.)**[a]	53.80±12.59	52.70±11.88	0.053	52.48 ±10.57	52.91 ±11.33	0.295	60.37 ±12.33	52.91 ±11.33	**<0.001**
**Age at menarche, years (mean ± s.d.)**[b]	15.51±1.95	16.09±1.93	**<0.001**						
**Age at natural menopause, years (mean ± s.d.)**[c]	48.47±4.76	49.11±4.26	**0.010**						
**Age at first live birth, years (mean ± s.d.)**[d]									
**Menopausal status**									
Premenopausal									
Natural menopausal									
Unnatural menopausal									
**Estrogen receptor (ER)**									
Positive									
Negative									
**Progesterone receptor (PR)**									
Positive									
Negative									

[a] Age information was available in 70 breast cancer cases in Discover stage, 3215 breast cancer cases and 2660 controls in Validation stage.
[b] Age at menarche information was available in 67 breast cancer cases in Discover stage, 3040 breast cancer cases and 2649 controls in Validation stage.
[c] Age at natural menopause age information was available in 40 breast cancer cases in Discover stage, 983 breast cancer cases and 1129 controls in Validation stage.
[d] Age at first live birth information was available in 64 breast cancer cases in Discover stage ,1812 breast cancer cases and 2236 controls in Validation stage.
[e] Menopause information was available in 70 breast cancer cases in Discover stage, 3080 breast cancer cases and 2637 controls in Validation stage.
[f] ER, PR and HER2 status information were available in 53, 50, and 43 breast cancer cases in Discover stage, 2852, 2841 and 1389 breast cancer cases in Validation stage, respectively.
[g] Hepatocellular carcinoma and colorectal cancer shared the cancer-free control samples.
[h] Gender information was available in 1095 colorectal cancers.

Supplementary Table 4. 48 variants obtained from *BRCA1/2* scanning in 70 familial breast cancer cases

Gene	Location(hg19)	Variant type	Nucleotide change	MAF (1000 Genomes Project)	Classification of ENIGMA[a]
BRCA1	chr17:41209081-41209081	synonymous	c.5560C>A	NA	Class 3
BRCA1	chr17:41215910-41215910	frameshift	c.5133del	NA	Class 3
BRCA1	chr17:41223094-41223094	missense	c.4837A>G	0.3558	Class 1
BRCA1	chr17:41223130-41223130	nonsense	c.4801A>T	NA	Class 5
BRCA1	chr17:41228529-41228529	missense	c.4460A>G	NA	Class 3
BRCA1	chr17:41234470-41234470	synonymous	c.4803T>C	0.371	Class 3
BRCA1	chr17:41244000-41244000	missense	c.3548A>G	0.371	Class 1
BRCA1	chr17:41244100-41244100	missense	c.3448C>T	0.004	Class 3
BRCA1	chr17:41244291-41244291	nonsense	c.3257del	NA	Class 5
BRCA1	chr17:41244435-41244435	missense	c.3113A>G	0.371	Class 1
BRCA1	chr17:41244936-41244936	missense	c.2612C>A	0.371	Class 3
BRCA1	chr17:41244982-41244982	missense	c.2566T>C	0.014	Class 1
BRCA1	chr17:41245237-41245237	synonymous	c.2311T>C	0.3353	Class 1
BRCA1	chr17:41245466-41245466	synonymous	c.2082C>T	0.3365	Class 1
BRCA1	chr17:41245587-41245587	frameshift	c.1961del	NA	Class 5
BRCA1	chr17:41245847-41245848	frameshift	c.1700dup	NA	Class 5
BRCA1	chr17:41246724-41246724	missense	c.824G>A	NA	Class 3
BRCA1	chr17:41246753-41246753	synonymous	c.795T>C	0.001	Class 1
BRCA1	chr17:41256140-41256140	missense	c.440del	NA	Class 3
BRCA1	chr17:41258487-41258487	synonymous	c.198T>C	NA	Class 2
BRCA1	chr17:41267763-41267763	synonymous	c.114G>A	0.0126	Class 1
BRCA2	chr13:32899249-32899249	missense	c.353G>A	NA	Class 3
BRCA2	chr13:32906480-32906480	missense	c.865A>C	0.0737	Class 1
BRCA2	chr13:32906729-32906729	missense	c.1114A>C	0.2494	Class 1
BRCA2	chr13:32906980-32906980	synonymous	c.1365A>G	0.0737	Class 1
BRCA2	chr13:32910452-32910452	missense	c.1960G>A	NA	Class 3
BRCA2	chr13:32910721-32910721	synonymous	c.2229T>C	0.095	Class 1
BRCA2	chr13:32910963-32910963	nonsense	c.2471T>G	NA	Class 5
BRCA2	chr13:32911463-32911463	missense	c.2971A>G	0.097	Class 1
BRCA2	chr13:32911516-32911516	synonymous	c.3257C>T	NA	Class 3
BRCA2	chr13:32911757-32911757	nonsense	c.3265C>T	NA	Class 5
BRCA2	chr13:32911888-32911888	synonymous	c.3396A>C	0.368	Class 1
BRCA2	chr13:32912299-32912299	synonymous	c.3807T>C	0.173	Class 1
BRCA2	chr13:32912699-32912699	missense	c.4207A>G	NA	Class 3
BRCA2	chr13:32912799-32912799	missense	c.4307T>C	NA	Class 3
BRCA2	chr13:32913055-32913055	synonymous	c.4563A>G	NA	Class 1
BRCA2	chr13:32913070-32913070	synonymous	c.4578A>C	0.001	Class 2
BRCA2	chr13:32914173-32914174	nonsense	c.5681dup	NA	Class 5
BRCA2	chr13:32915005-32915005	synonymous	c.6513G>C	NA	Class 1
BRCA2	chr13:32929083-32929083	missense	c.7093C>A	NA	Class 3
BRCA2	chr13:32929140-32929140	nonsense	c.7149T>A	NA	Class 3
BRCA2	chr13:32929232-32929232	synonymous	c.7242A>G	0.369	Class 1
BRCA2	chr13:32929399-32929400	frameshift	c.7409dup	NA	Class 5
BRCA2	chr13:32930651-32930651	missense	c.7522G>A	NA	Class 3
BRCA2	chr13:32937526-32937526	missense	c.8187G>T	0.012	Class 1
BRCA2	chr13:32930598-32930598	missense	c.7469T>C	0.003	Class 1
BRCA2	chr13:32972388-32972388	synonymous	c.9738C>A	NA	Class 2
BRCA2	chr13:32972884-32972884	missense	c.10234A>G	0.021	Class 1

[a] The ENIGMA classification was ascertained from BRCA exchange.

Supplementary Table 5. No recurrent BRCA1/2 germline variants in Validation stages

Gene	Nucleotide change	Validation stage							
		Breast Cancer		Cervical Cancer		Hepatocellular Carcinoma		Colorectal Cancer	
		Cases (N = 3217)	Controls (N = 2660)	Cases (N = 1133)	Controls (N = 1098)	Cases (N=2044)	Controls* (N=1116)	Cases (N = 1116)	Controls* (N=1116)
BRCA1	c.5133delA	0/3217	0/2660	0/1133	0/1098	0/2044	0/1116	0/1116	0/1116
BRCA1	c.4460A>G	0/3217	0/2660	0/1133	0/1098	0/2044	0/1116	0/1116	0/1116
BRCA1	c.3448C>T	0/3217	0/2660	0/1133	0/1098	0/2044	0/1116	0/1116	0/1116
BRCA1	c.1961del	0/3217	0/2660	0/1133	0/1098	0/2044	0/1116	0/1116	0/1116
BRCA1	c.1700dup	0/3217	0/2660	0/1133	0/1098	0/2044	0/1116	0/1116	0/1116
BRCA1	c.824G>A	0/3217	0/2660	0/1133	0/1098	0/2044	0/1116	0/1116	0/1116
BRCA2	c.353G>A	0/3217	0/2660	0/1133	0/1098	0/2044	0/1116	0/1116	0/1116
BRCA2	c.1960G>A	0/3217	0/2660	0/1133	0/1098	0/2044	0/1116	0/1116	0/1116
BRCA2	c.2471T>G	0/3217	0/2660	0/1133	0/1098	0/2044	0/1116	0/1116	0/1116
BRCA2	c.3265C>T	0/3217	0/2660	0/1133	0/1098	0/2044	0/1116	0/1116	0/1116
BRCA2	c.4207A>G	0/3217	0/2660	0/1133	0/1098	0/2044	0/1116	0/1116	0/1116
BRCA2	c.5681dup	0/3217	0/2660	0/1133	0/1098	0/2044	0/1116	0/1116	0/1116
BRCA2	c.7093C>A	0/3217	0/2660	0/1133	0/1098	0/2044	0/1116	0/1116	0/1116
BRCA2	c.7149T>A	0/3217	0/2660	0/1133	0/1098	0/2044	0/1116	0/1116	0/1116
BRCA2	c.7522G>A	0/3217	0/2660	0/1133	0/1098	0/2044	0/1116	0/1116	0/1116

***BRCA1* c.5133delA**

Case.26

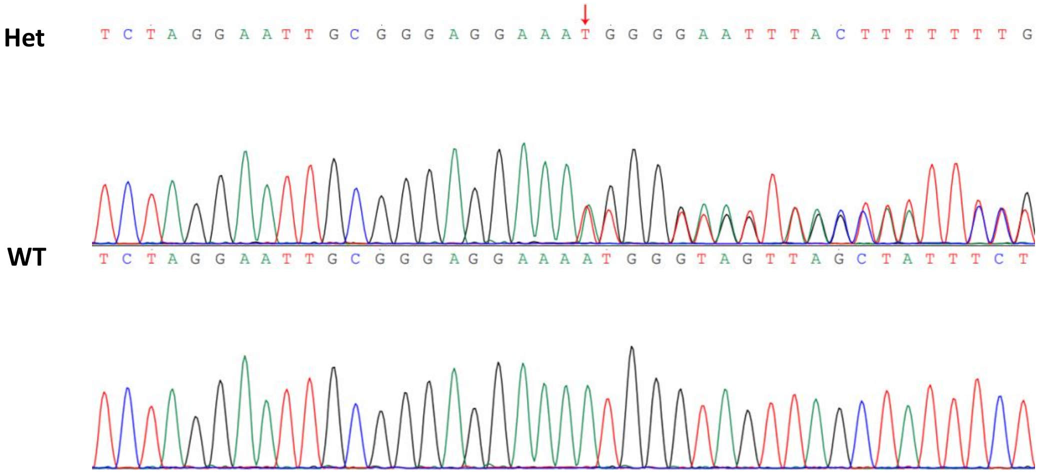

***BRCA1* c.4801A>T**

Case.5

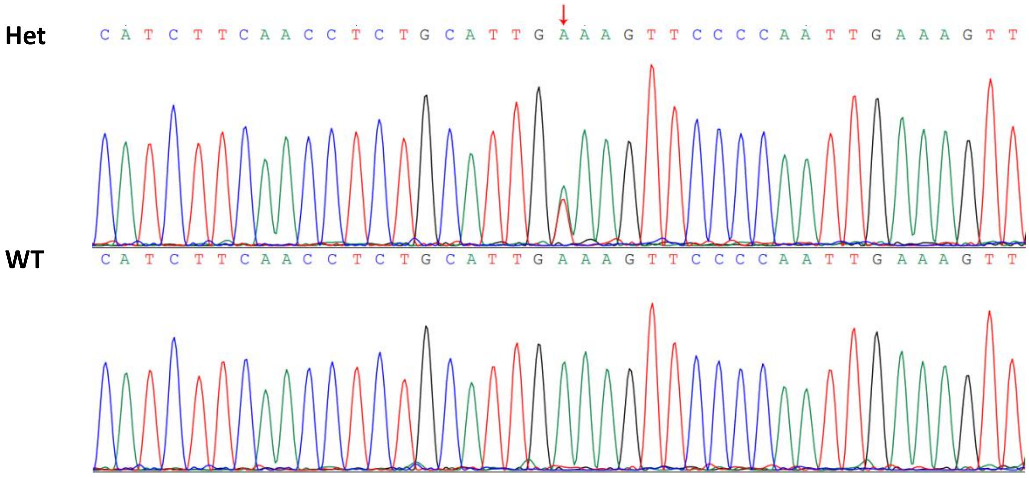

Case.71

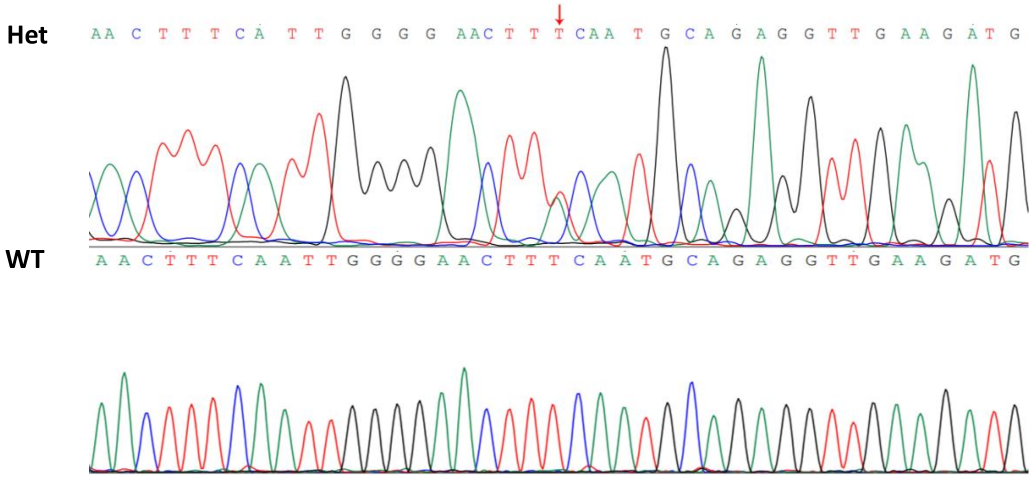

Case.78

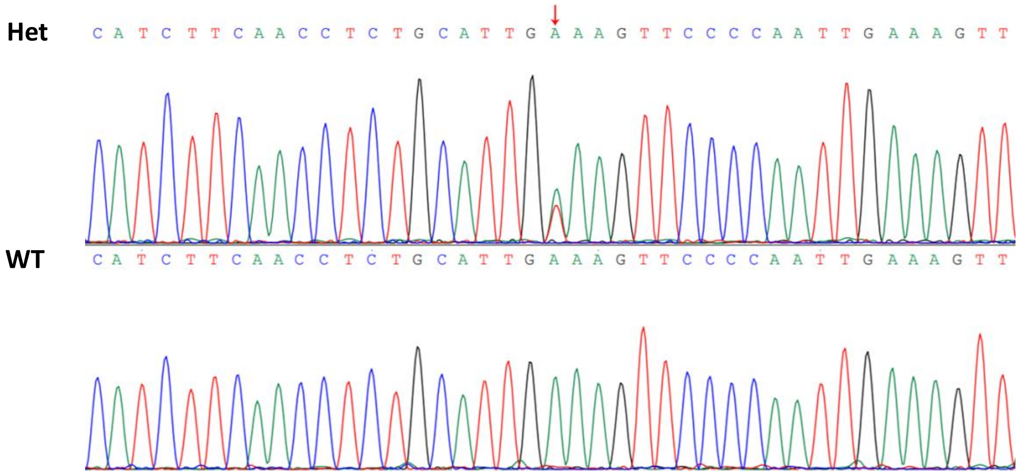

***BRCA1* c.4460A>G**

Case.21

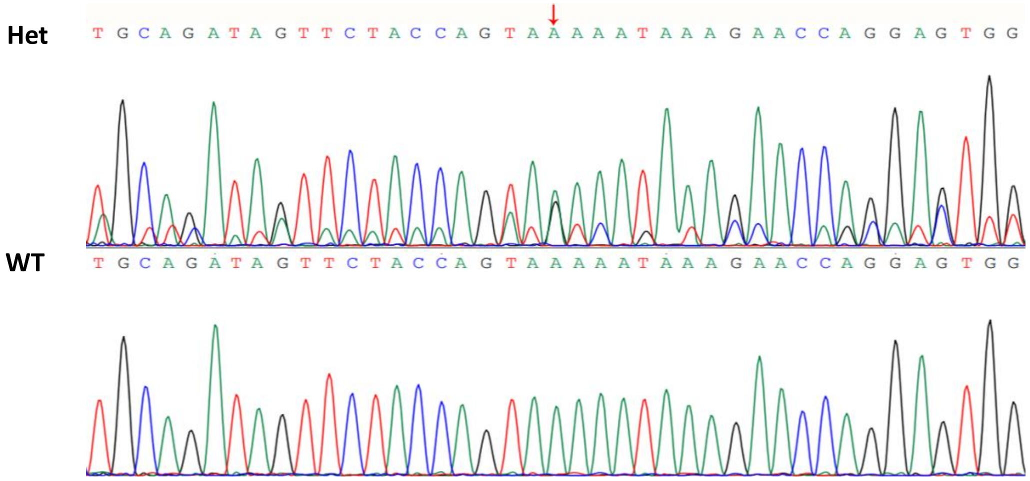

Case.23

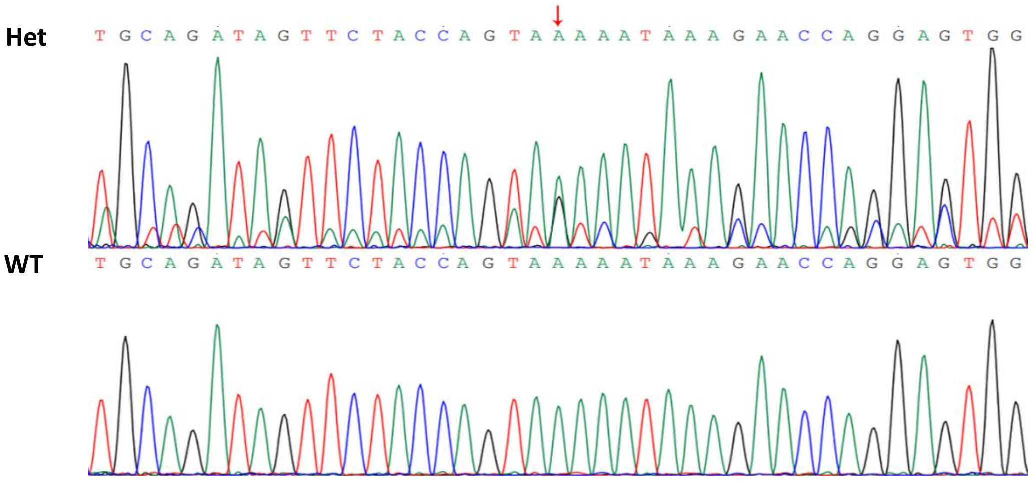

***BRCA1* c.3448C>T**

1-14-12

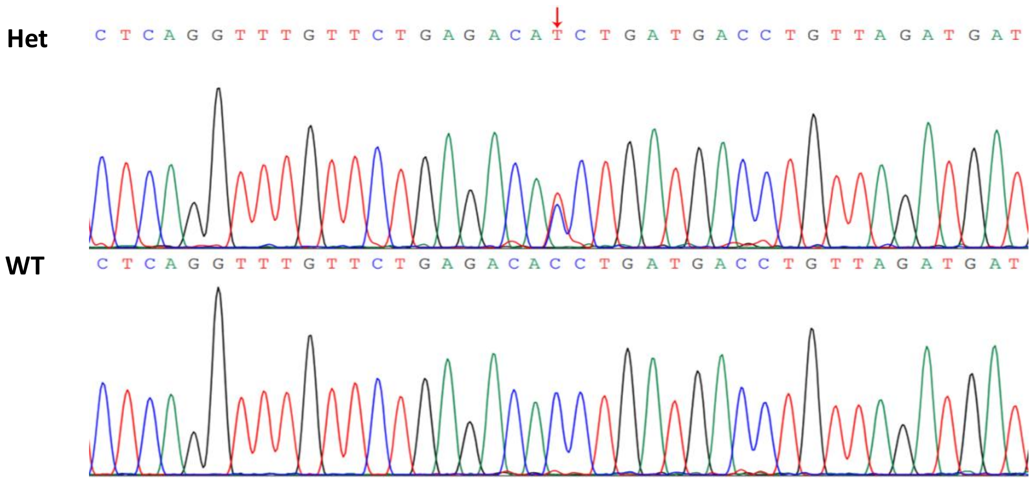

***BRCA1* c.3257delT**

Case.62

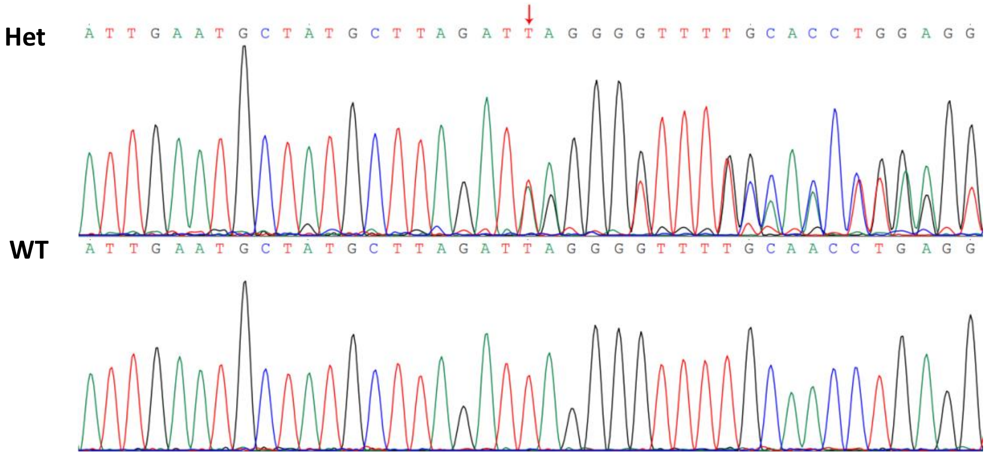

Case.72

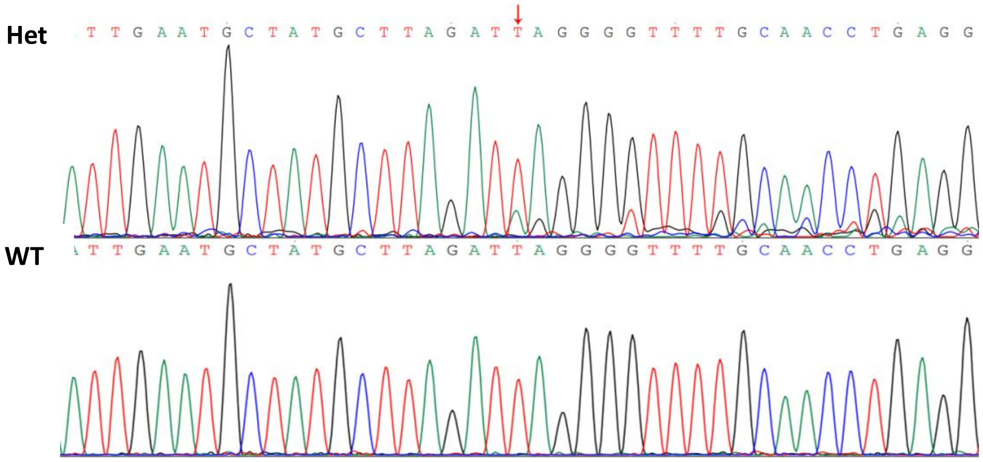

Case.73

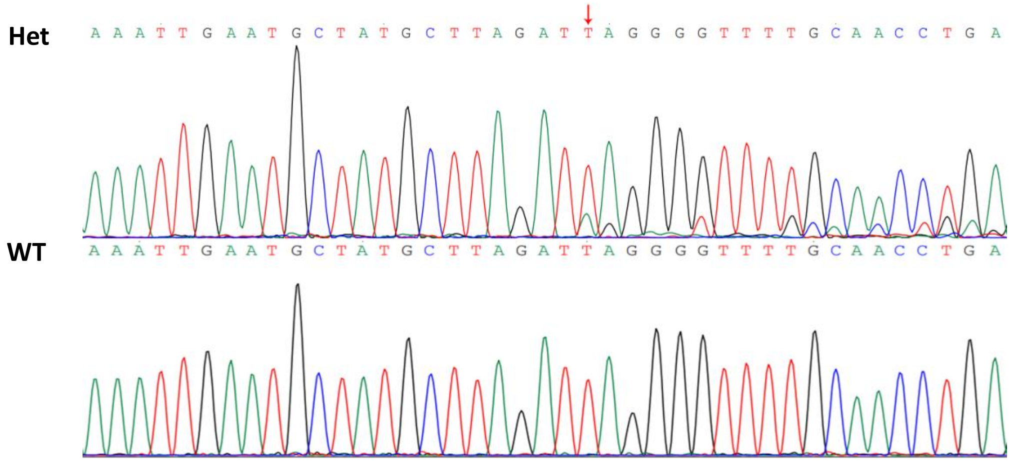

Case.74

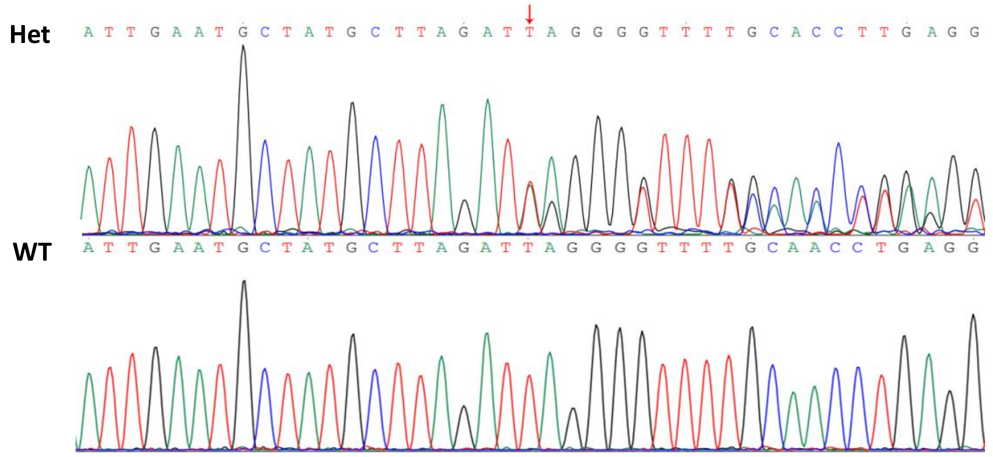

BRCA1 c.2566T>C

Case.7

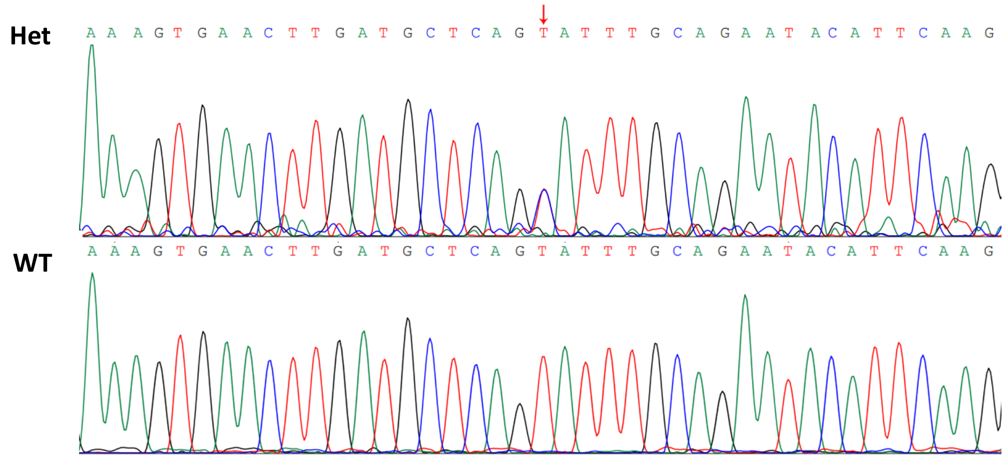

Case.29

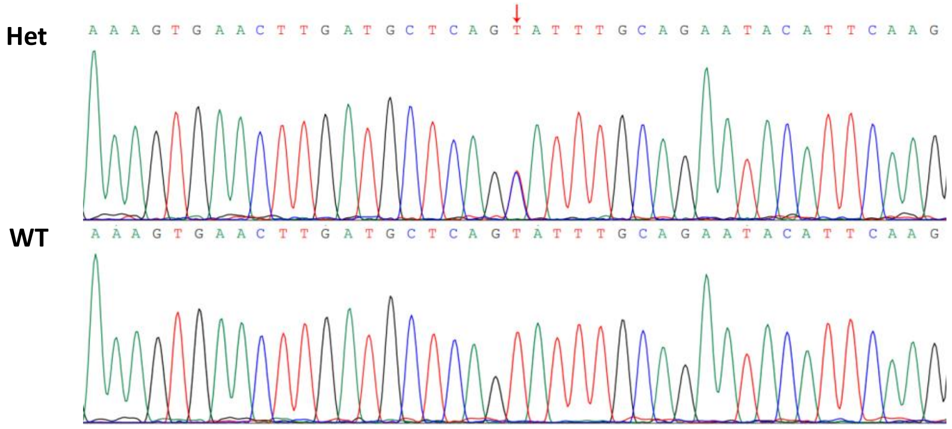

Case.63

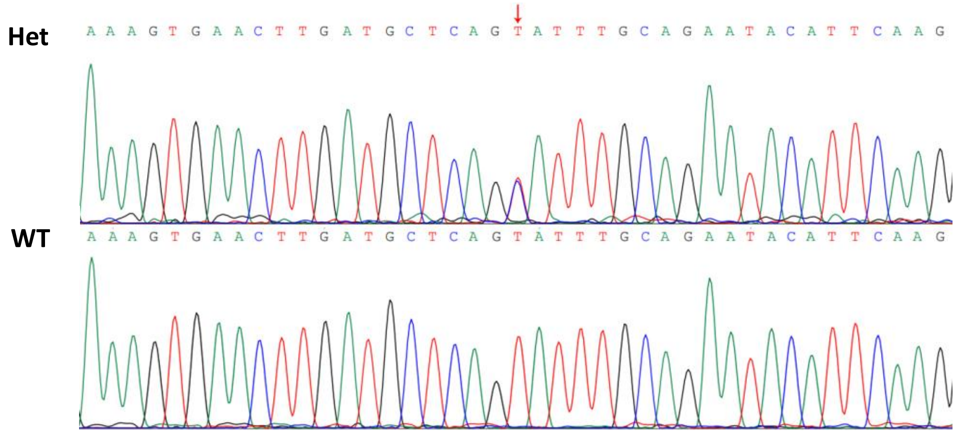

Case.66

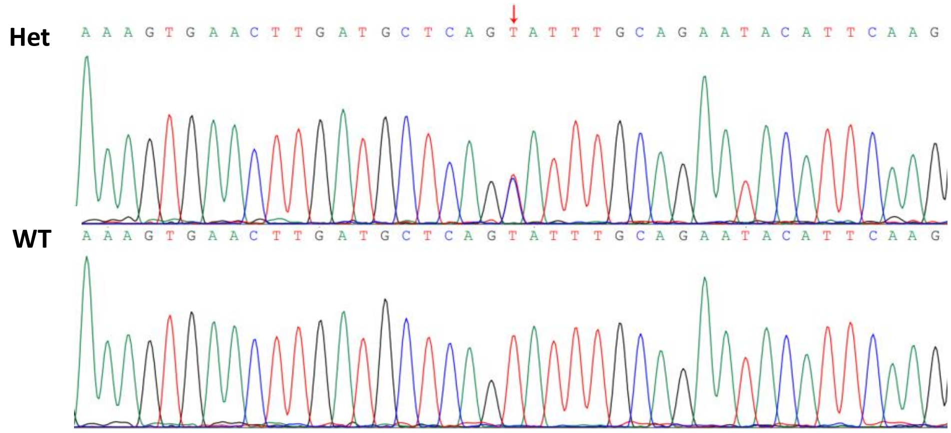

***BRCA1* c.1961delA**
Case.67

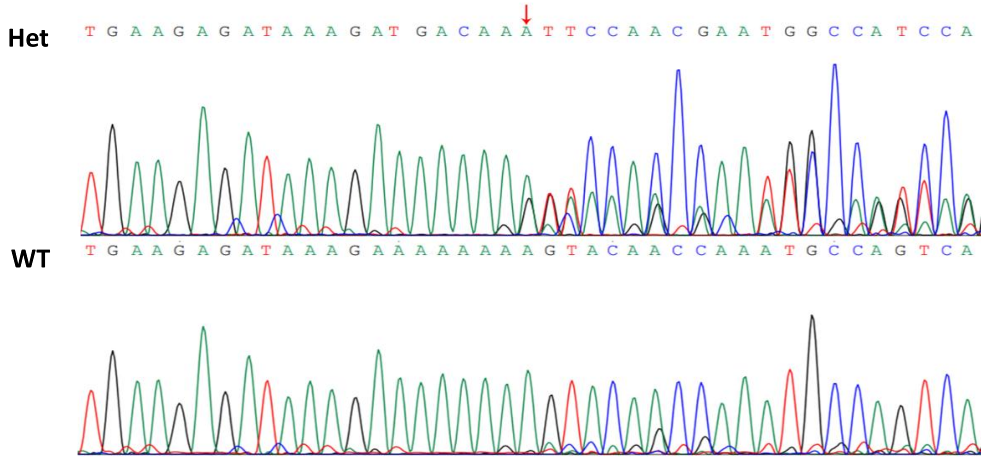

***BRCA1* c.1700_1701insA**
Case.40

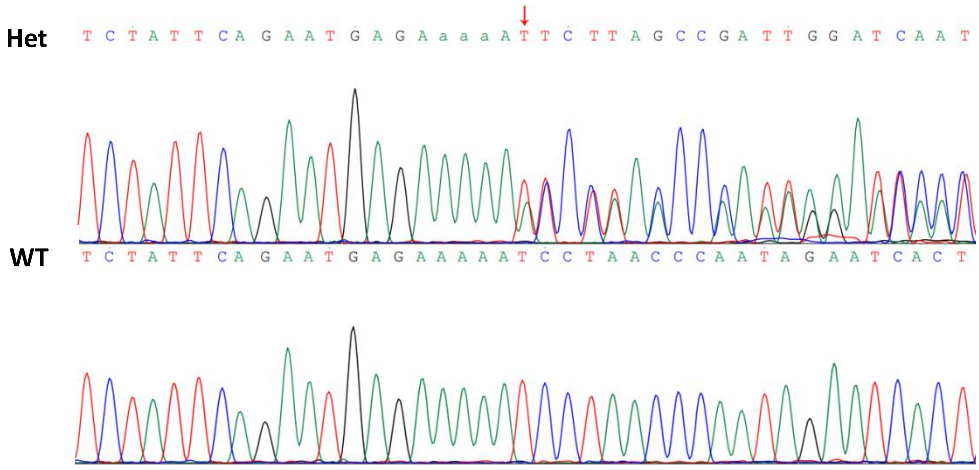

***BRCA1* c.440delT**
Case.50

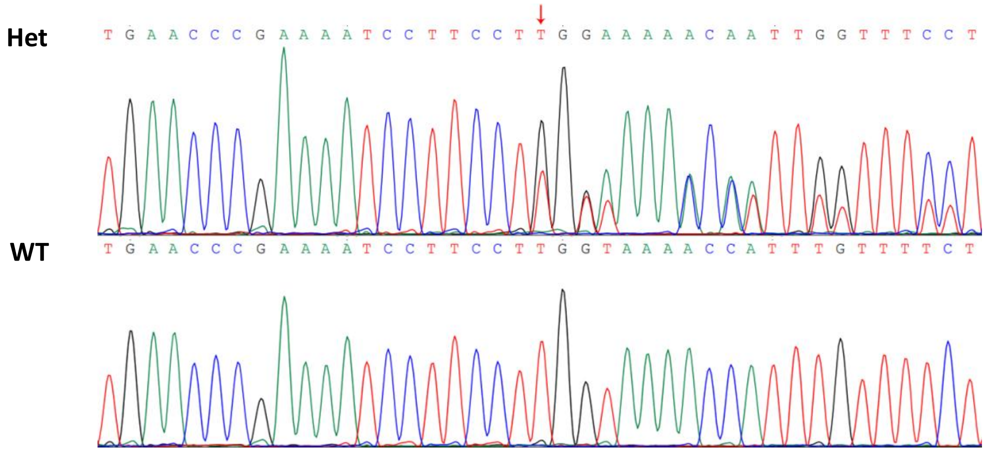

Case.75

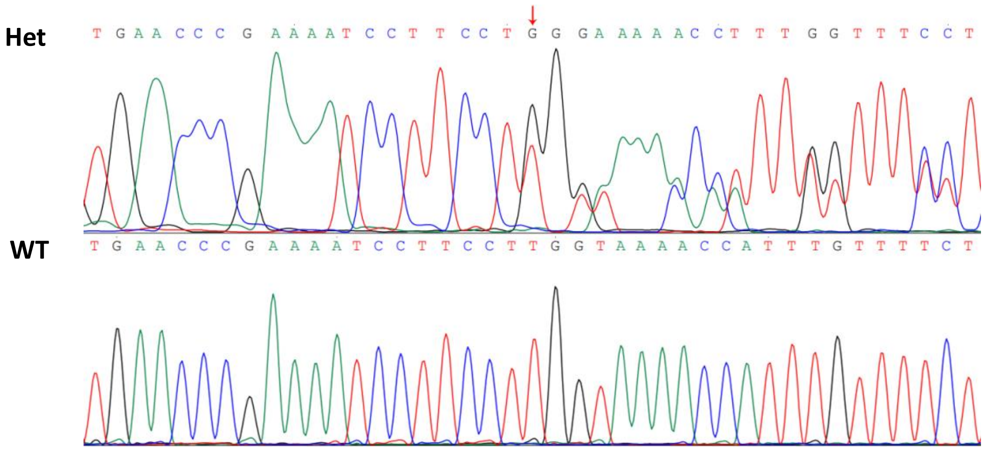

BRCA1 **c.824G>A**

Case.4

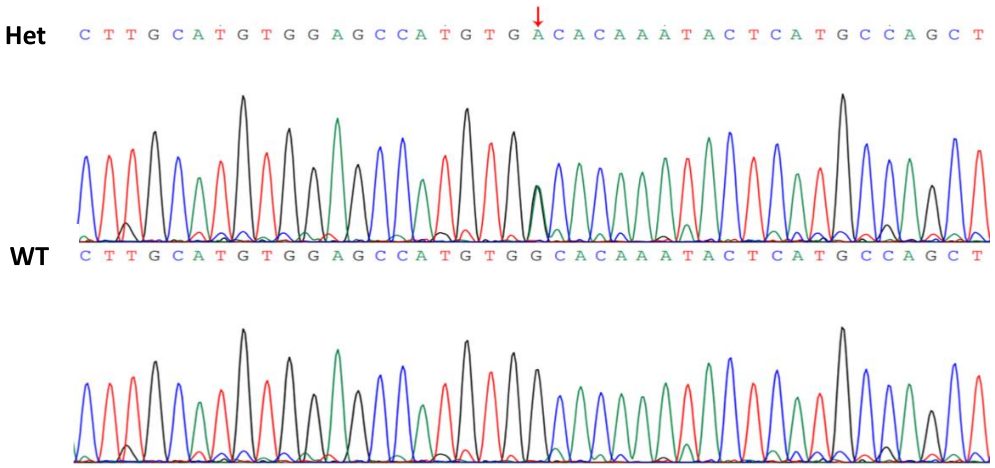

Case.70

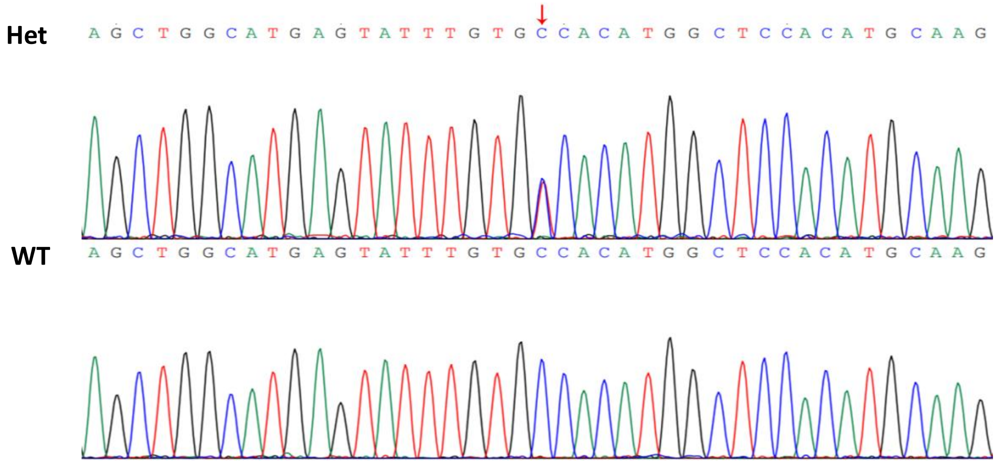

***BRCA2* c.353G>A**

Case.37

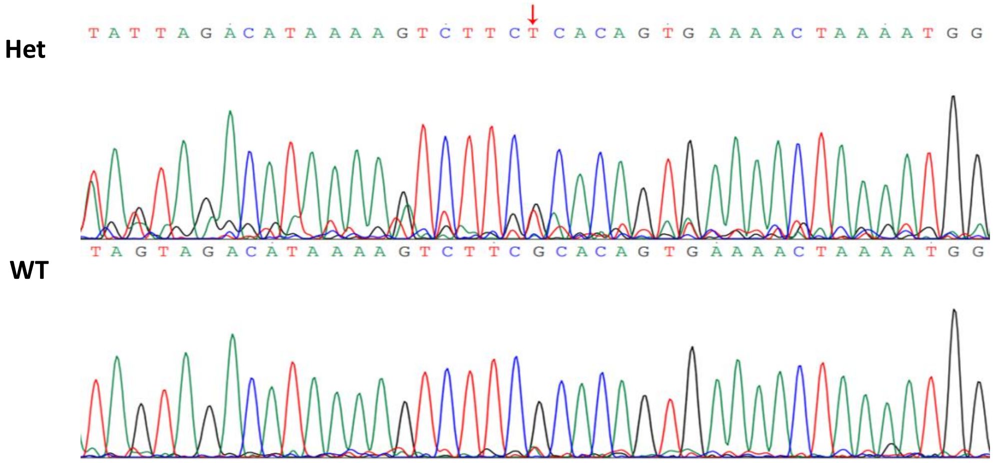

***BRCA2* c.1960G>A**

Case.25

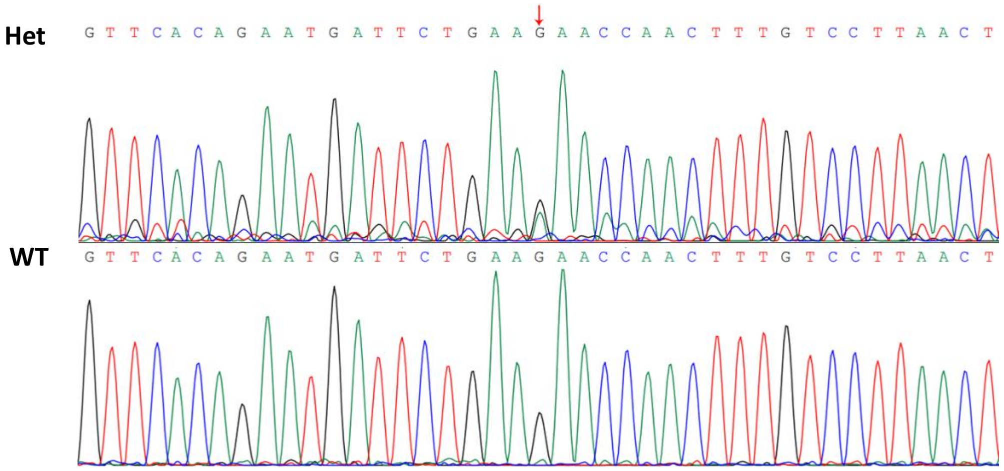

***BRCA2* c.2471T>G**

Case.10

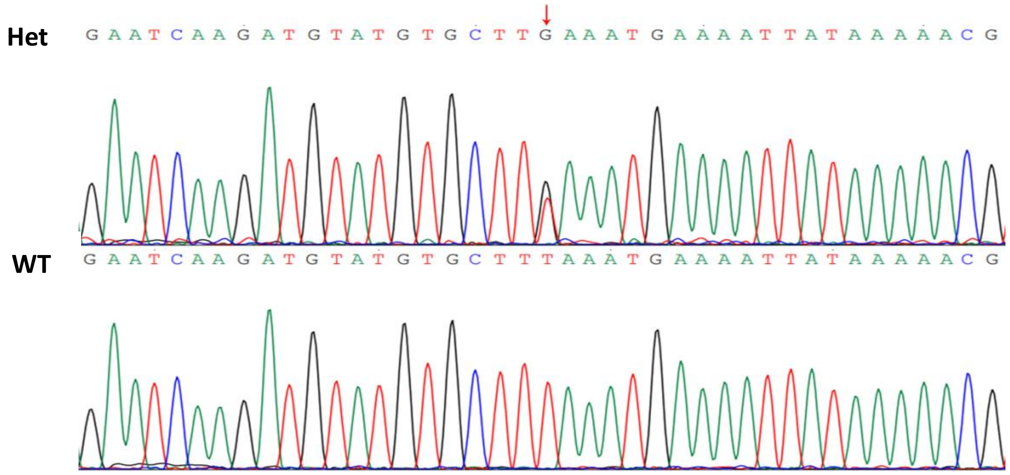

***BRCA2* c.3265C>T**

Case.57

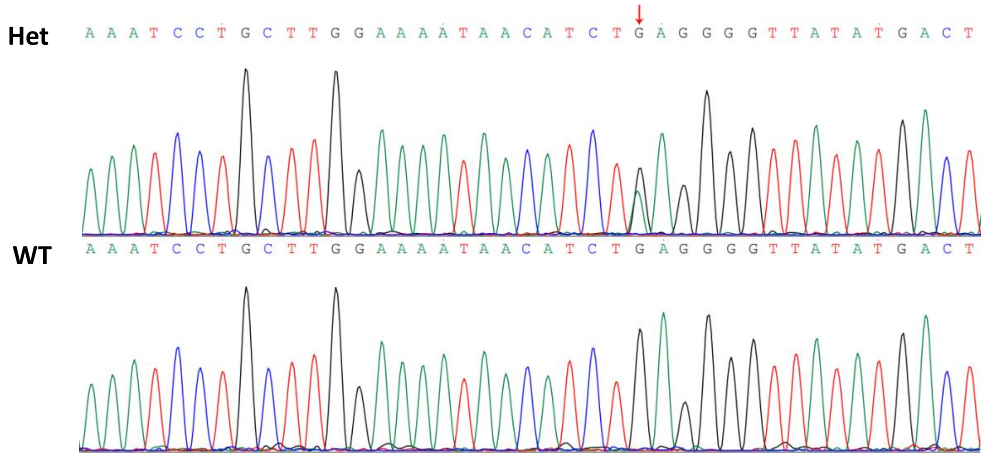

***BRCA2* c.4207A>G**

Case.35

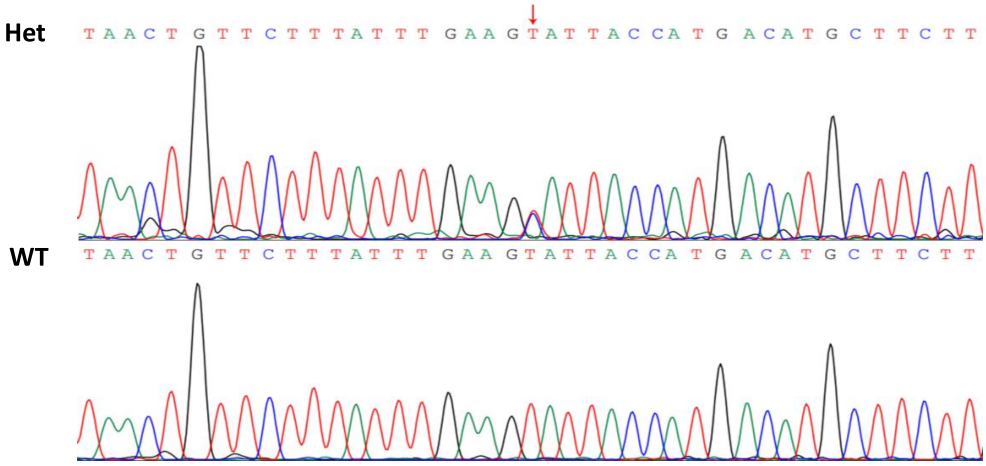

***BRCA2* c.4307T>C**

Case.64

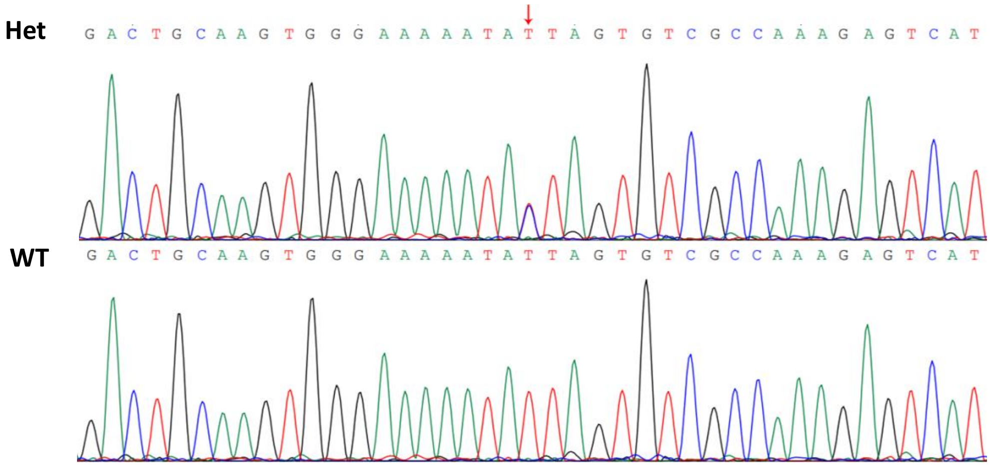

Case.79

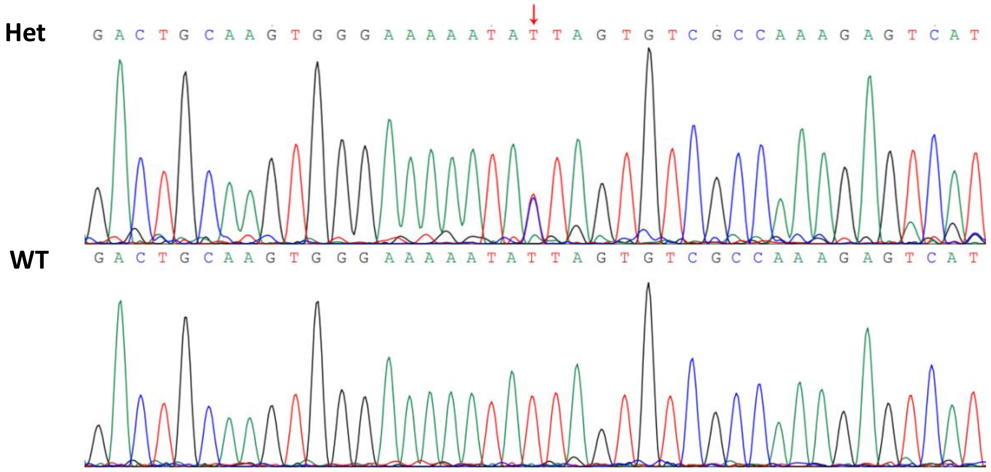

***BRCA2* c.5681dupA**

Case.61

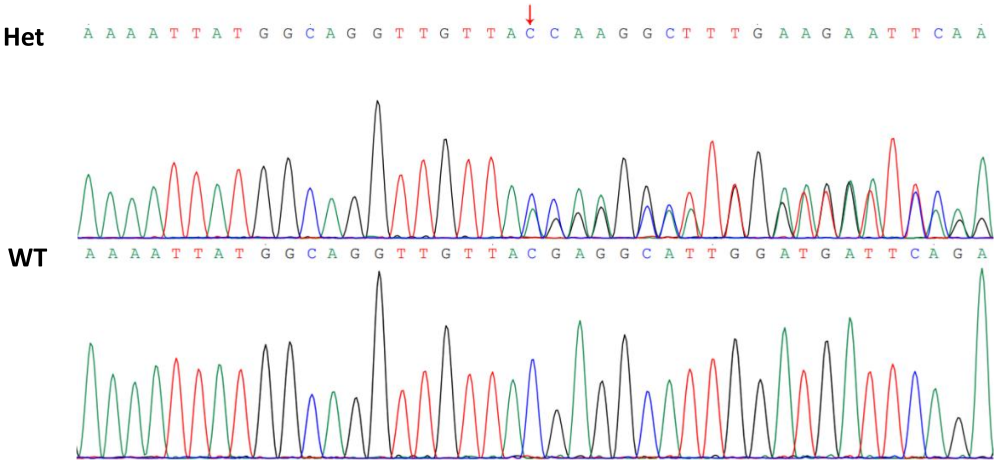

***BRCA2* c.7093C>A**

Case.26

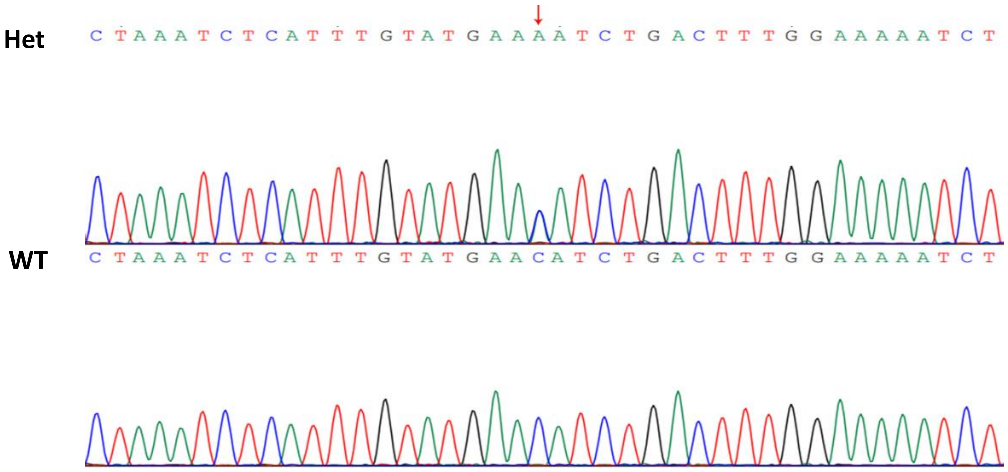

***BRCA2* c.7149T>A**

Case.69

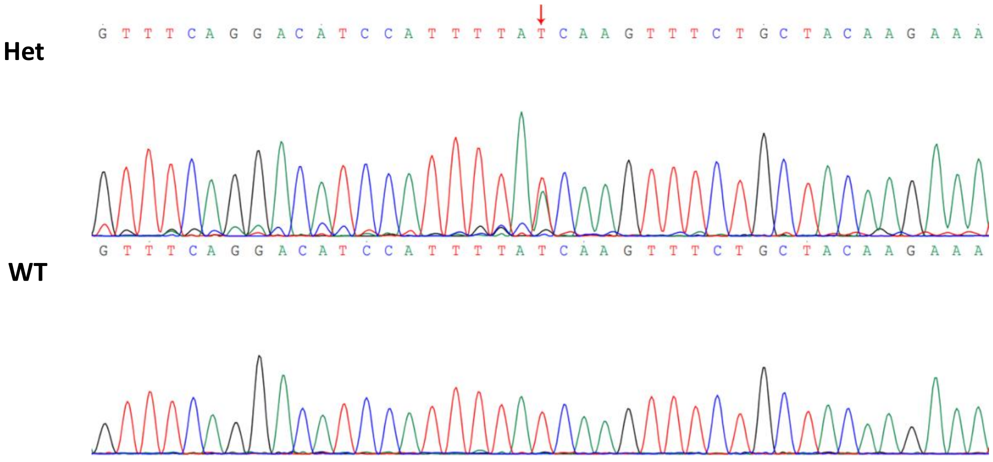

***BRCA2* c.7409dupT**

Case.18

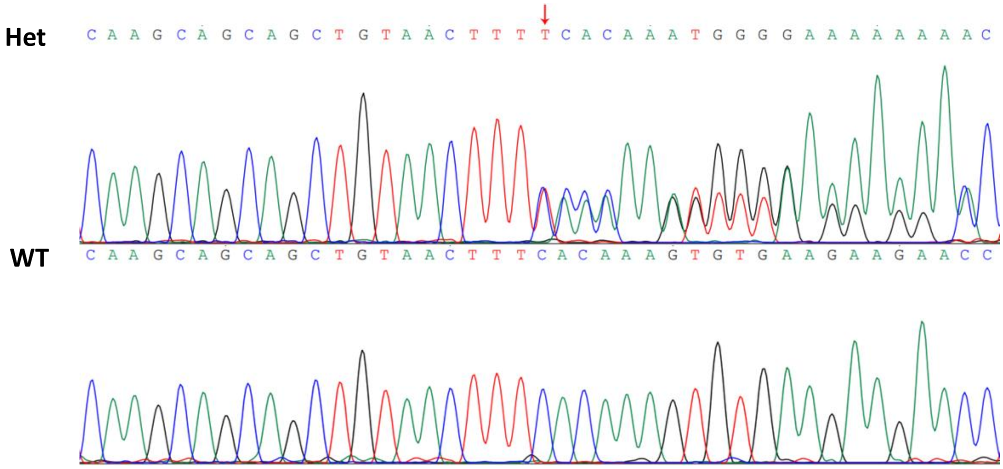

Case.76

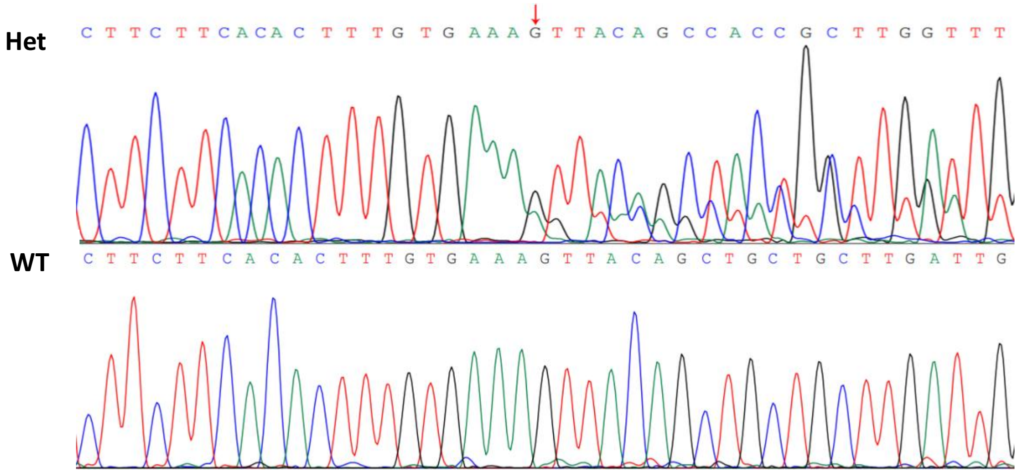

Case.77

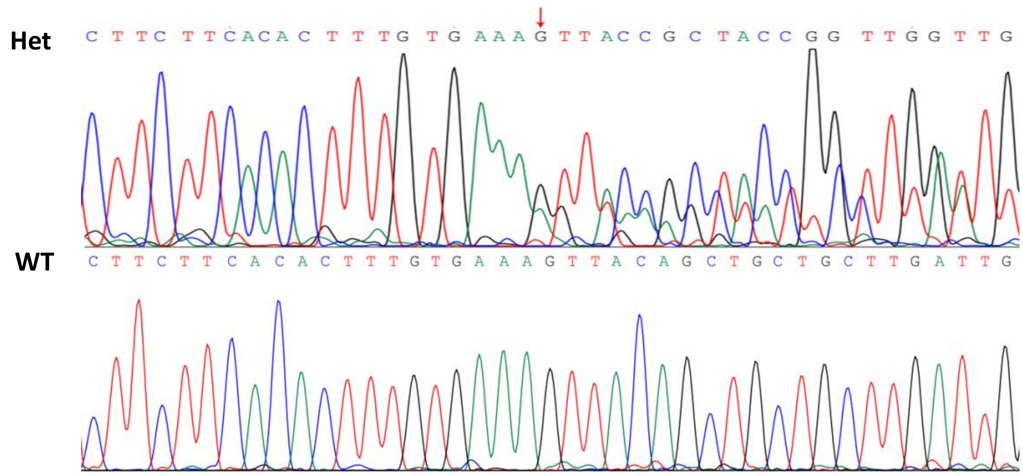

Case.80

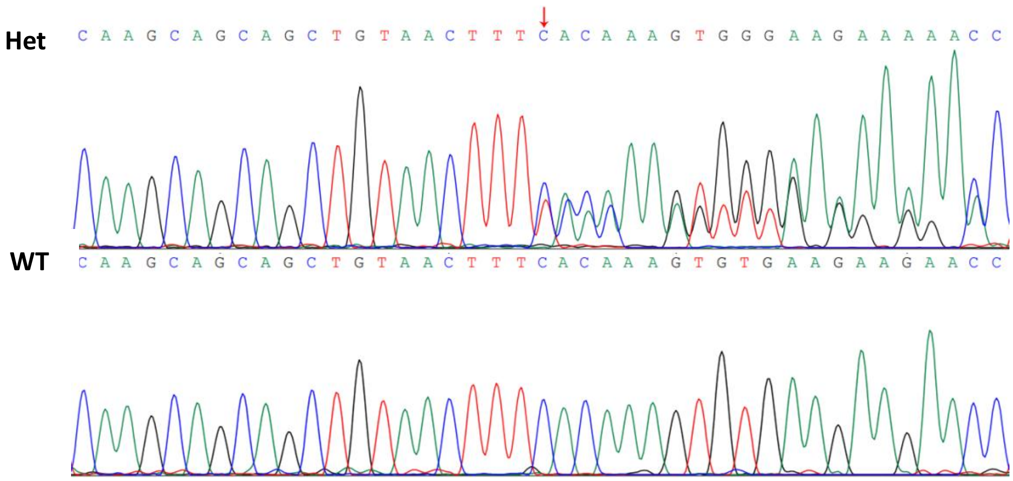

***BRCA2* c.7522G>A**

Case.3

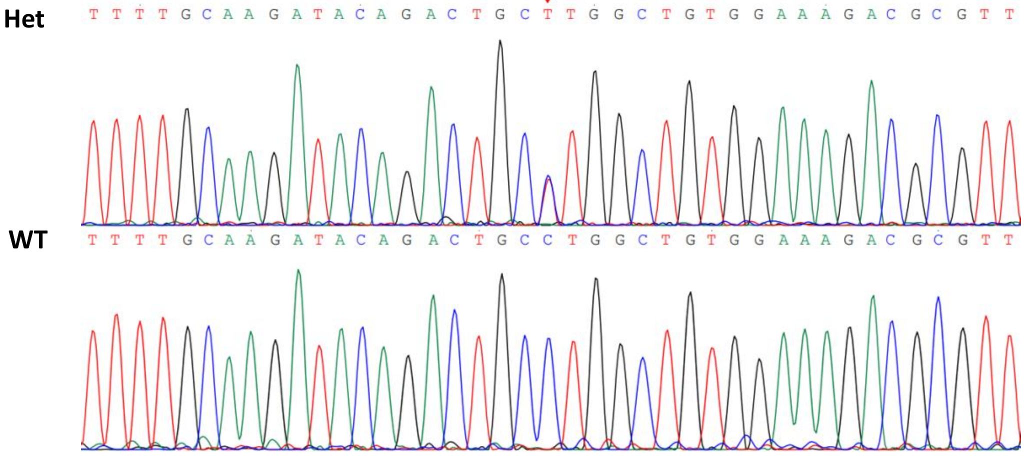

***BRCA2* c.8414G>T**

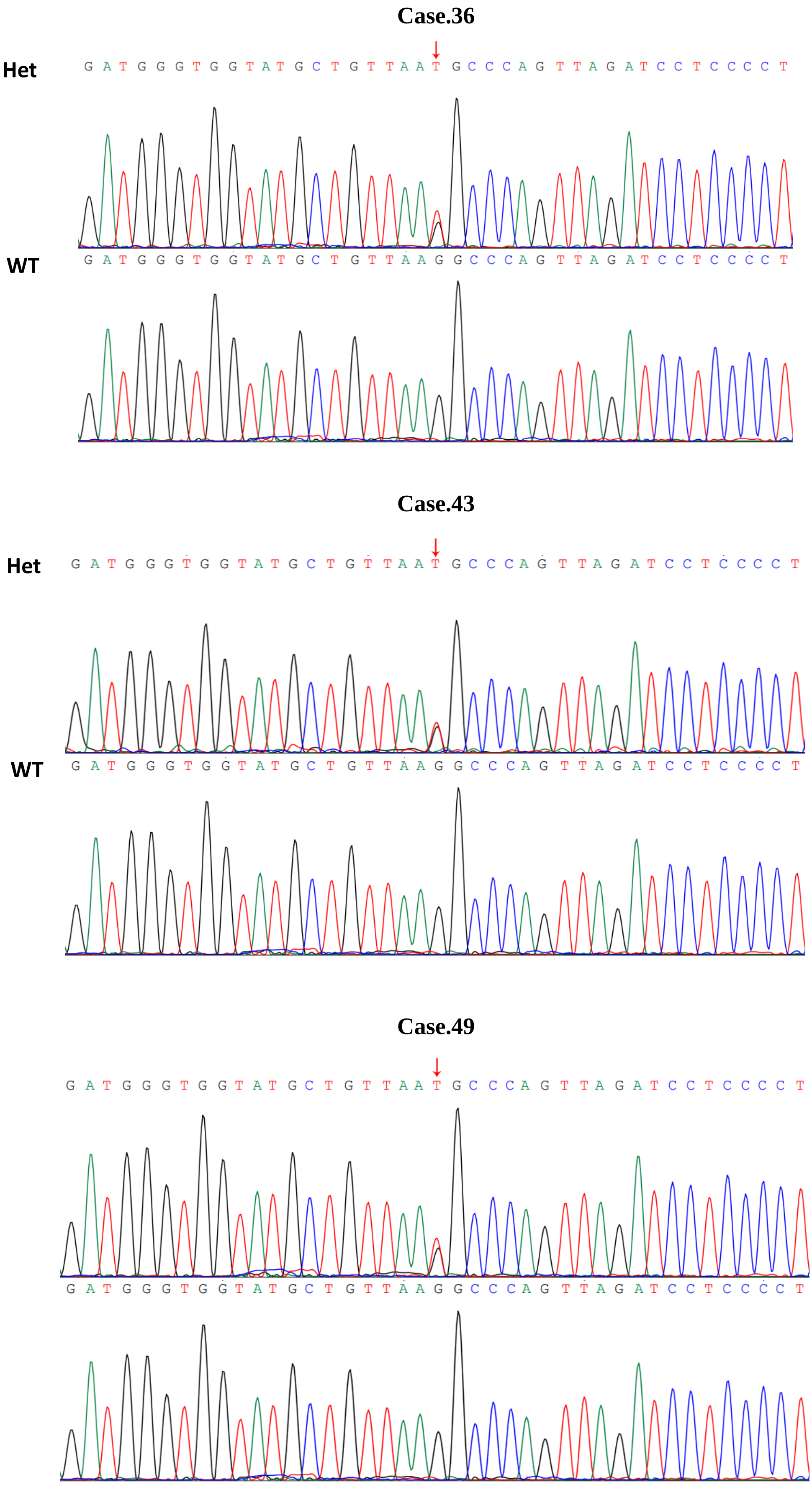

Supplementary Figure 2

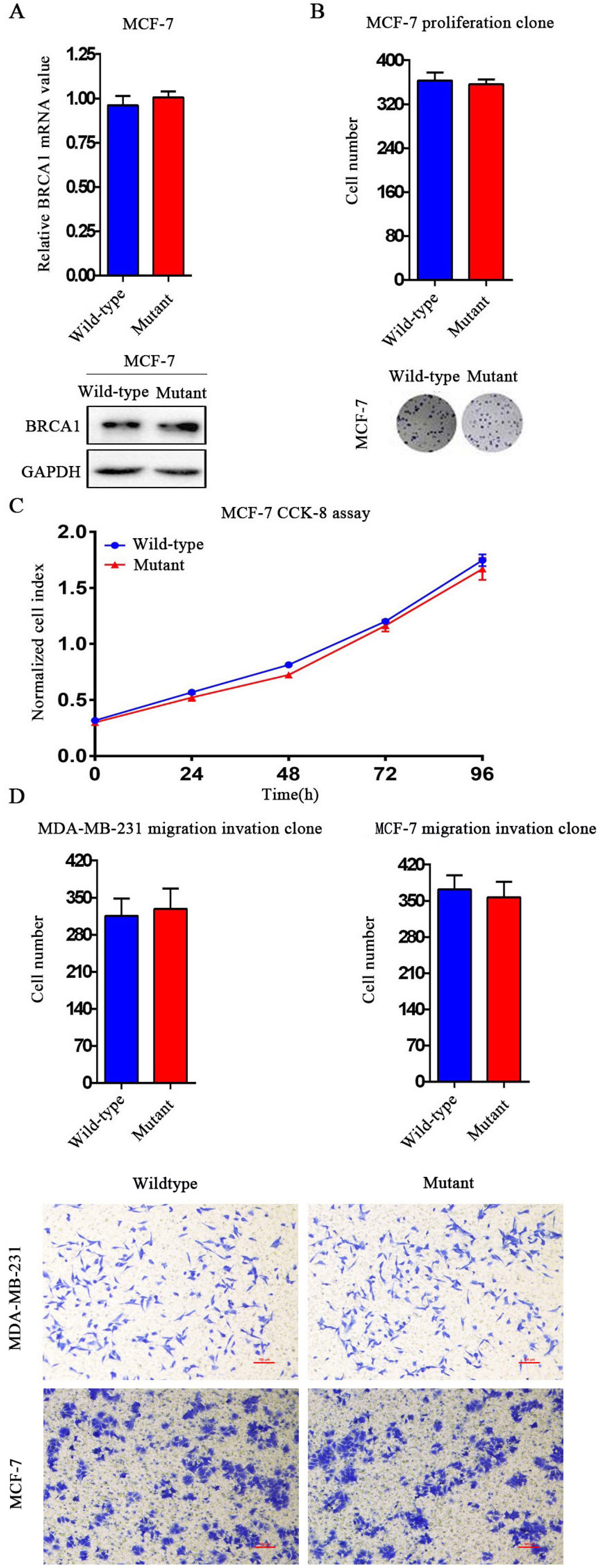

Supplementary Figure 3

A

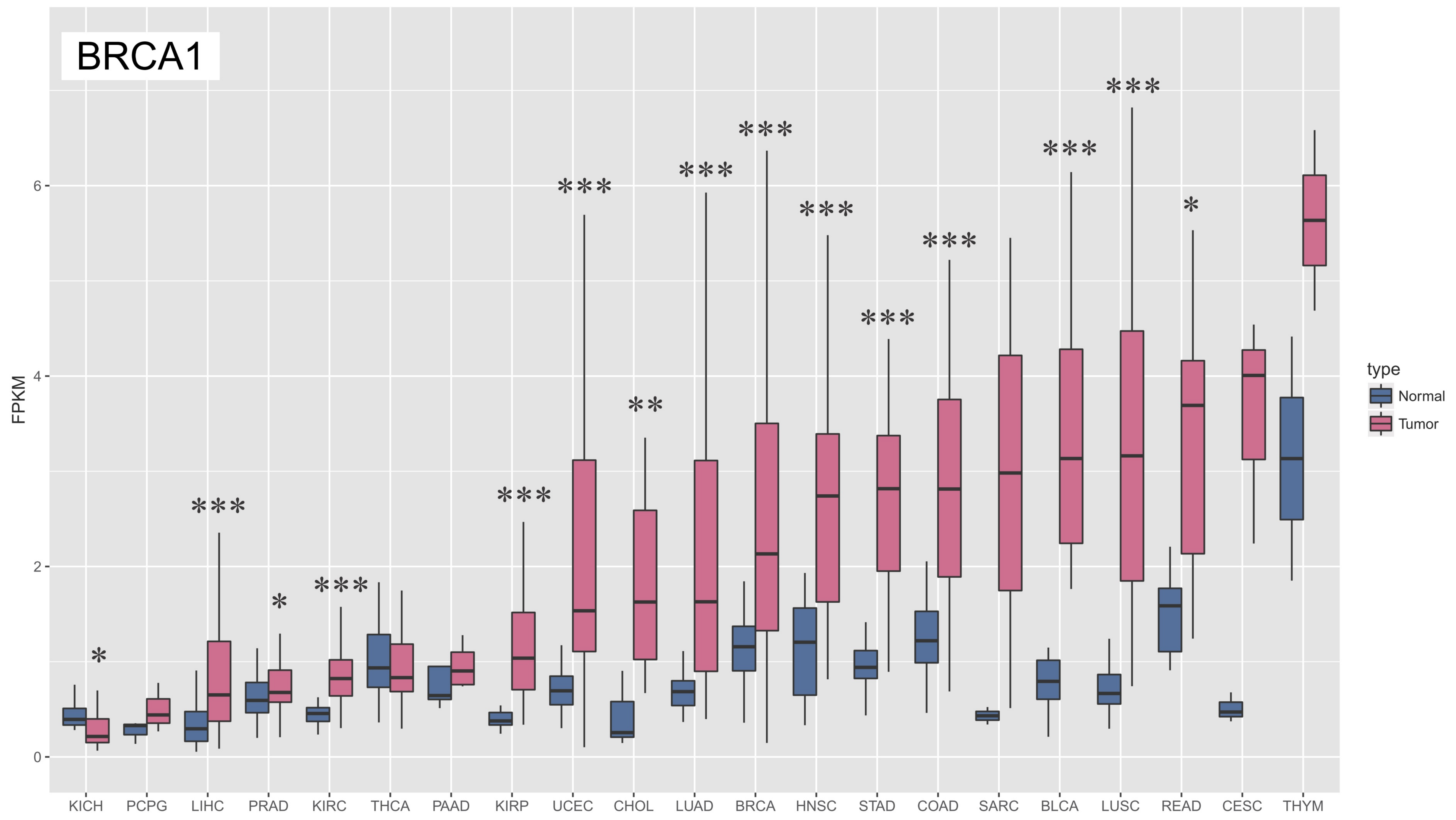

B

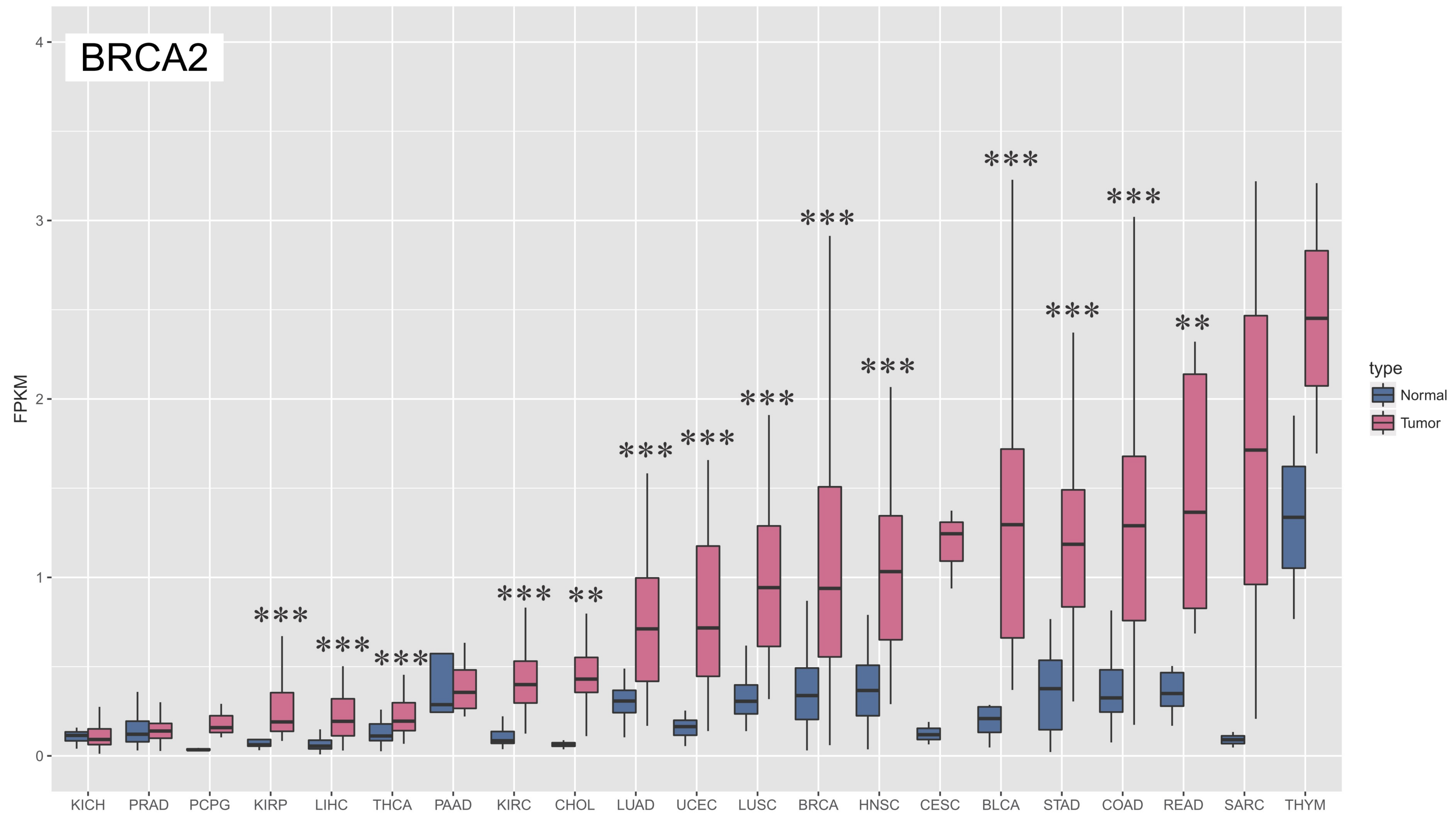

Supplementary Table 1. Information of primers for *BRCA1*.

Supplementary Table 2. Information of primers for *BRCA2*.

Supplementary Table 3. Distributions of selected variables in multi-cancer cases and cancer-free controls.

Supplementary Table 4. 48 variants obtained from *BRCA1/2* scanning in 70 familial breast cancer cases.

Supplementary Table 5. No recurrent *BRCA1/2* germline variants in validation stages.

Supplementary Figure 1. Sequence results of 20 detected variants in 31 cases in *BRCA1* and *BRCA2*.

Supplementary Figure 2. (A) The mRNA level of MCF-7 transfected with different plasmids showed no significant difference in different groups ($P > 0.05$). Western blot analysis showed that the *BRCA1* proteins translated by *BRCA1* c.3257del were not truncated in MCF-7 cells. (B) The colony formation efficiency of MCF-7 transfected with *BRCA1* c.3257del was not increased ($P > 0.05$). (C) *BRCA1* c.3257del-overexpressing MCF-7 cell proliferation efficiency was not increased as measured by the CCK-8 assay in vitro ($P > 0.05$). (D) Both MDA-MB-231 and MCF-7 cell migration were measured by transwell assay, and no significant difference in cell migration was detected ($P > 0.05$).

Supplementary Figure 3. *BRCA1* and *BRCA2* expression data from TCGA in multiple tumors compared with paired normal tissues.
